# Systematic discovery about NIR spectral assignment from chemical structural property to natural chemical compounds

**DOI:** 10.1038/s41598-019-45945-y

**Published:** 2019-07-01

**Authors:** Lijuan Ma, Yanfang Peng, Yanling Pei, Jingqi Zeng, Haoran Shen, Junjie Cao, Yanjiang Qiao, Zhisheng Wu

**Affiliations:** 1 Beijing University of Chinese Medicine, Beijing, 102488 China; 2 Key Laboratory of TCM-information Engineering of State Administration of TCM, Beijing, 102488 China; 3 Beijing Key Laboratory for Basic and Development Research on Chinese Medicine, Beijing, 102488 China; 4 Hubei University of Chinese Medicine, Hubei, 430065 China; 5 Fujian University of Traditional Chinese Medicine, Fujian, 350122 China

**Keywords:** Infrared spectroscopy, Drug development

## Abstract

Spectra-structure interrelationship is still the weakness of NIR spectral assignment. In this paper, a comprehensive investigation from chemical structural property to natural chemical compounds was carried out for NIR spectral assignment. Surprisingly, we discovered that NIR absorption frequency of the skeleton structure with sp^2^ hybridization is higher than one with sp^3^ hybridization. Specifically, substituent was another vital factor to be explored, the first theory discovery demonstrated that the absorption intensity of methyl substituted benzene at 2330 nm has a linear relationship with the number of substituted methyl C-H. The greater the number of electrons given to the substituents, the larger the displacement distance of absorption bands is. In addition, the steric hindrance caused by the substituent could regularly reduce the intensity of NIR absorption bands. Furthermore, the characteristic bands and group attribution of 29 natural chemical compounds from 4 types have been systematic assigned. These meaningful discoveries provide guidance for NIR spectral assignment from chemical structural property to natural chemical compounds.

## Introduction

Near-infrared (NIR) spectroscopy is fast in process, intact to sample and friendly to environment^1^. As a process analytical technology (PAT), it is widely applied to the qualitative and quantitative analysis in agriculture and food industry^2–5^. At present, its application in the field of medicine is still a hot topic. Li *et al*. applied NIR spectroscopy to two traditional Chinese medicine (TCM) technical processes control^6^, and proposed a non-destructive method to analyze Compound E Jiao oral liquid^7^. However, the components of Chinese materia medica (CMM) are complex. The accuracy and robustness of NIR model are critical for its industrial application. For more robust model, Zhou *et al*. applied the boosting partial least square (PLS) regression for a better model performance^8^. Filgueiras *et al*. used synergy interval support vector regression (siSVR) to select effective variables and obtained a more robust model^9^. Pan *et al*. found that backward interval PLS was the appropriate method for the particle size model, while synergy interval PLS was the optimal method for the lobetyolin model^10^. Preview works demonstrated that variable selection is indeed necessary for the NIR application in CMM^11,12^.

However, the variables in these studies, which are selected through chemometric method, lacked practical significance and interpretability for the material structure. Many teams^13–15^ have found that using different chemometric methods to process the same data set, the selected variables vary greatly. The absorbance of NIR is mainly controlled by the stretching vibration overtones and combination modes of hydrogen-containing groups (X-H) including O-H, N-H, C-H, S-H, etc.^16^. Hence, to make the predictive models more robust and interpretive, it is essential to assign the spectra based on the interrelation between spectra and structure^17^. Recently, it is delighted to see that there are certain sporadic reports about these. The C-H and O=C hydrogen bonding were studied to explore the isothermal crystallization kinetics of poly (3-hydroxybutyrate)^18^. Czarnecki *et al*. reported a great deal of researches on the relationship between spectra and structure^19–21^. They summarized a large number of NIR literature and emphasized the significance of interpretation of spectra-structure interrelationship for spectral assignment^22^. Our previous researches also illustrated that spectral assignment was of great significance for NIR modeling. The NIR spectral assignments of Yunkang Oral Liquid^23^ and *Lonicera japonica* have been performed and made the models more accurate^24^.

Spectral assignment methods include chemometric, quantum mechanical calculation, two-dimensional correlation spectroscopy, and so on. The second derivative (2nd) spectra^25^ and difference spectra (DS)^26^ can overcome the deficiency of the original spectrum to some extent. They are mainly used to eliminate the noise of the original spectrum and enhance the spectral resolution. Mathian *et al*. analyzed the detection limit of the major types of hypogene phyllosilicates by the NTR 2nd spectra^27^. Gezici *et al*. pointed out the model performance of the characteristic band selected by the DS method was significantly better than that of the full spectrum^28^.

Comparatively speaking, quantum mechanical method can quickly obtain the attribution of the characteristic group by combining the experimental NIR spectra and the theoretical frequency^29^. DMol_3_ is the fastest quantum mechanical calculation method for molecular density functional theory (DFT) due to its unique advantages in handling electrostatics^30^. The DFT calculation under the Materials Studio Dmol3 library have been used to investigate the structure, thermodynamics and chemical properties of the C-120-nanostructure modified with Ti atoms^31^. Verissimo *et al*. also applied the DFT calculations to conduct a study on the structure of ethambutol^32^.

Simultaneously, for complex compounds, the one-dimensional spectra mentioned above have limitation for further spectra-structure analysis^33^. The two-dimensional correlation spectrum (2D-COS) was extended to “Generalized Two-Dimensional Correlation Analysis” by Noda^34^. 2D-COS can assign more small and weak bands, which are masked by the autocorrelation peaks on the two-dimensional scale^35^. The molecular structures (especially the hydrogen bond) of methanol and ethanol have been analyzed by NIR and 2D-COS^36^. Two-dimensional correlation analysis was carried out to investigate the variation of sugars and water involved in the osmo-dehydration process^37^. The experimental 2D-COS patterns have been successfully reproduced to monitor the effects of conformational isomerism to the shape of NIR spectra^38^. 2D-COS coupled with Fourier transform infrared the changes of different organic constituents in landfill leachate were tracked in Fenton oxidation processes^39^. Recently, Noda has further proposed the two-trace two-dimensional (2T2D) correlation and discussed its potential application^40^.

Unfortunately, these spectra-structure studies usually focus on hydrogen-bonding^41^ and the interaction of intermolecular^42^, although some about micro-heterogeneity^43^. Chemical structural properties, such as the hybridization type of X (C, N, O, and S), the presence or absence of compound substituents, and the steric hindrance, have not been explored. In addition, the influence of solvent is a tough challenge in application of spectral assignment methods and needs to be discussed. Moreover, the spectral assignment of complex natural chemical compounds is necessary but insufficient, such as mosses, phenylpropanoids, alkaloids and steroids^44^.

Accordingly, deuterated reagent was used to explore the effects of key factors of specific compounds with chemical structural properties, including atomic hybridization mode, quantity and position of substituents, and steric hindrance. Besides, the identification of characteristic variables was conducted by experiments and theoretical calculations. In addition, characteristic variables of mosses, phenylpropanoids, alkaloids and steroids have been explored. Furthermore, in order to get more detailed spectra-structure information, 2D-COS has been proposed for group attribution with concentration as the interference term. From chemical structural property to natural chemical compounds, this paper provides a systematic and valuable discovery for NIR spectral assignment.

## Results

### Near infrared (NIR) spectral assignment of hybridization type

There are differences in the NIR characteristic bands of substances with different structures, and the hybridization type is a critical factor that affects the diversity in absorption. Supplementary Fig. S1 shows the NIR raw spectrum of benzene and cyclohexane, from which, the third overtone absorption of the methylene in cyclohexane at 890 nm, the second overtone absorption at 1210 nm, the second combination mode at 1400 nm, and the first overtone absorption peak at 1760 nm has been assigned.

Another discovery is that the C-H of backbone structure of benzene has the third overtone, the second overtone, and the first overtone absorption respectively at 880 nm, 1140 nm, and 1660 nm. These results indicate that the absorption frequency of C with sp^2^ hybridization (benzene) is higher than one with sp^3^ hybridization (cyclohexane).

**Figure 1:**
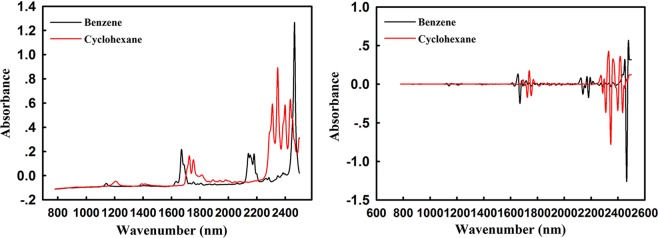
The NIR raw spectra and 2nd spectra of benzene and cyclohexane with same molarity. There were obvious absorption differences in overtone region between benzene and cyclohexane have been presented. In addition, benzene has a series of absorption peaks at 2130 nm and 2460 nm.

In order to exclude the difference in absorption intensity caused by the uneven number of molecules, further studies were carried out using the same molar concentration of benzene and cyclohexane. The NIR raw spectra and 2nd spectra of benzene and cyclohexane with same molarity were shown in Fig. 1, in which, obvious absorption differences in overtone region between benzene and cyclohexane have been presented.

We also found that benzene has a series of absorption peaks related to C-H and C-C stretching vibrations in the combination mode 2130 nm and single-strong absorption peak caused by C-H stretching and bending vibration at 2460 nm. In terms of cyclohexane, there were four strong absorption peaks centering 2400 nm, which was the result of combination mode of C-H stretching and bending vibration. The results demonstrated that the C-H absorption frequency of benzene is higher than one of cyclohexane with a smaller bond force constant, proving again that the carbon atom with sp^2^ hybridization type had a larger absorption frequency. The assignment of hybridization type is significantly meaningful theory for a wider range of application of NIR spectroscopy both in production and clinical diagnosis.

**Figure 2:**
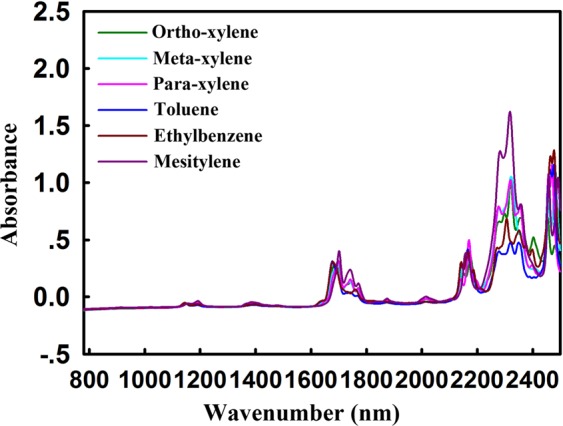
NIR raw spectra of carbon tetrachloride with 1 mol/L toluene, xylene, ethylbenzene, and mesitylene. There was a strong absorption peak centered at 2330 nm.

Owing to the same hybridization type and skeleton structure, the identification of homologues is another difficulty in spectra-structural analysis. The NIR raw spectra of carbon tetra chloride solutions of different methyl-substituted benzene were displayed in Fig. 2. It is noteworthy that the absorption peak of the benzene ring skeleton C-H is not covered by the methyl C-H absorption, but its absorption intensity ratio changes. Additionally, a strong absorption peak centered at 2330 nm appears in the NIR spectra of methyl-substituted benzene, at where the absorption peak is the combined frequency absorption of methyl stretching vibration. This exploration provided another effective variable for identification of methyl substitution.

### NIR spectral assignment about the effects of the substitutes on core structure

For further quantitative research, the absorption intensity corresponding to the absorption peaks of benzene and its methyl substitutes at 1670 nm, 2130 nm, 2330 nm and 2460 nm were calculated and shown in Table 1. Accidentally, we spied out that at 2330 nm, the absorption intensity ratio of the toluene, xylenes, and mesitylene is about 1:2:3.5. For ethylbenzene, the absorption intensity at 2330 nm is between toluene and xylene. These indicate that the absorption intensity of methyl substituted benzene at 2330 nm is linear with the number of substituted methyl C-H. Briefly, as the number of carbon-hydrogen bonds increases, the NIR absorption intensity increases proportionally. This seminal discovery provides the theoretical basis of NIR quantitative for the first time.

**Table 1:** The absorption intensity corresponding to the absorption peaks of benzene and its methyl substitutes at 1670 nm, 2130 nm, 2330 nm, and 2460 nm.

Compounds	Absorbance			
	1670 nm	2130 nm	2330 nm	2460 nm
Benzene	0.4410	0.3746	—	2.0124
Ortho-xylene	0.2851	0.3598	1.0287	0.6920
Meta-xylene	0.2926	0.3456	1.0546	1.0973
Para-xylene	0.3156	0.4980	1.0237	1.1597
Toluene	0.3122	0.4169	0.4775	1.1594
Ethylbenzene	0.3082	0.3963	0.6867	1.2853
Mesitylene	0.4017	0.3846	1.6218	1.0621

**Figure 3:**
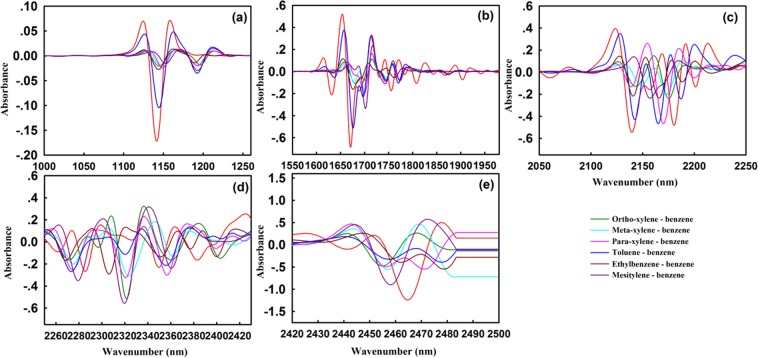
The partially enlarged NIR 2nd spectra of benzene and toluene, xylene, ethylbenzene, and mesitylene. Figure (**a**) showed two strong absorption peaks at 1138 nm and 1190 nm, respectively. In Figure (**b**), the absorption peak of the benzene ring skeleton at 1670 nm moved to the long wavelength direction by 30 nm. Figure (**c**) showed four sets of absorption peaks centered at 2150 nm. Figure (**d**) showed the absorption peak of the methyl group in the combined frequency region, in which, there is no regularity. Figure (**e**) showed a set of methyl absorption peaks centered at 2460 nm.

Furthermore, in-depth research of the differences in homologues have been carried out for more accurate quantitative analysis. Figure 3 displayed the partially enlarged NIR 2nd spectra of benzene, toluene, xylene, ethylbenzene, and mesitylene. The absorption peaks of the benzene ring skeleton C-H stretching vibration at 1138 nm and 1670 nm shifted to the long-wave direction due to the substitution of the methyl group, and the largest displacement was caused by mesitylene with 10 nm and 30 nm, respectively. Interestingly, the order of displacement of these two absorption peaks has a certain regularity, which is toluene ≈ ethylbenzene < ortho-xylene < meta-xylene < para-xylene < mesitylene. This major finding demonstrated that the inductive effect of the electron donating substituent made the benzene ring positively charged, inducing the displacement. In brief, the greater the number of electrons donating substituent, the larger the displacement distance of absorption peak is.

**Figure 4:**
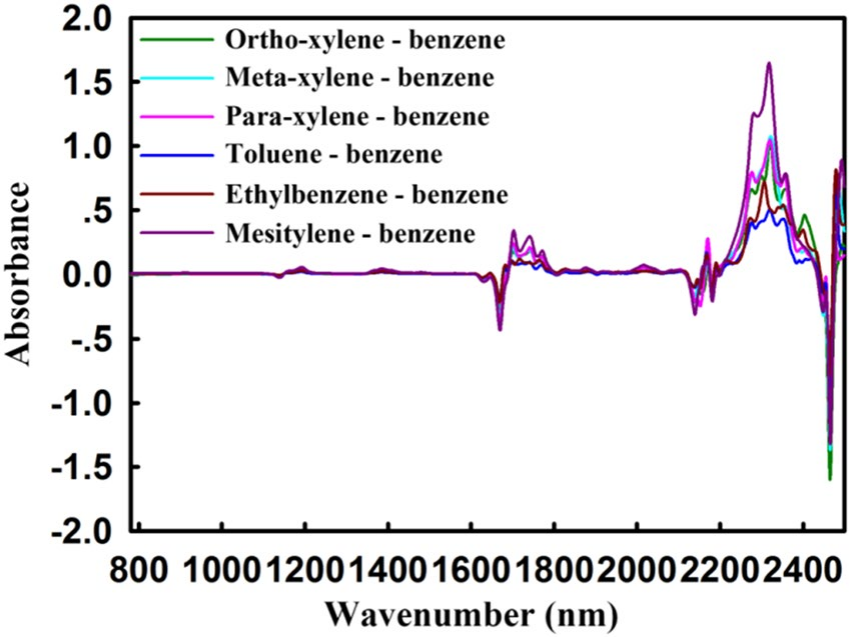
NIR DS between methyl substituted benzene and benzene. Compared with benzene, xylene, toluene, ethylbenzene and mesitylene had lower absorption intensities at 2460 nm, 2150 nm, 1670 nm and 1138 nm, and conversely higher at 1190 nm, 1400 nm, 1760 nm, 2330 nm and 2480 nm.

Compared to the 2nd method, the difference spectrum can react more quickly and directly to the effects of substituents on the basic structure. Figure 4 shows NIR DS between methyl substituted benzene and benzene. Clearly, for benzene ring C-H of toluene, xylene, ethylbenzene, and mesitylene, the absorption values of combination mode at 2460 nm and 2150 nm, the first overtone at 1670 nm and the second overtone at 1138 nm were negative.

In contrast, for the methyl C-H, the absorption values of combination mode at 2330 nm and 2480 nm, the second combination mode at 1400 nm, the first and second overtone at 1760 nm and 1190 nm were all positive. All these wavelengths reflect the difference between the toluene, xylene, ethylbenzene, and mesitylene. The partially enlarged DS of methyl substituted benzenes and benzene was shown in Supplementary Fig. S2. Strikingly, the orders of the overtone intensity of methyl C-H at 2330 nm and benzene C-H at 1138 nm, 1670 nm and 2130 nm, especially in the range of 780–2040 nm, all were ethylbenzene ≈ methylbenzene < xylene < mesitylene. The NIR DS between methyl substituted benzenes and toluene (see Supplementary Fig. S3) also matched this result. This was consistent with the result 2nd spectra, proving again the influence regularity of substituted methyl.

**Figure 5:**
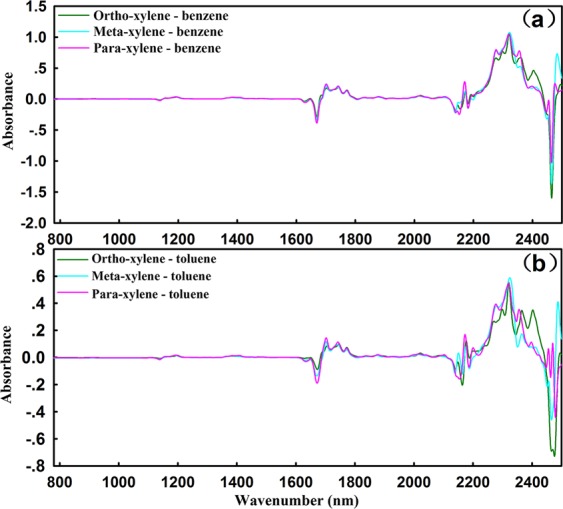
(**a**) The NIRS DS between xylenes and benzene; (**b**) The NIRS DS between xylenes and toluene. The absorption intensity at 2330 nm decrease from 1.0 in Figure (**a**) to 0.5 in Figure (**b**).

The symmetry of chemical structure is a pivotal factor affecting the NIR absorption intensity of substances. For further research, the NIRS DS of xylenes - benzene, and xylenes - toluene have been displayed in Fig. 5, and the enlarged views were shown in Supplementary Figs S4 and S5 for details. It is worth noting that the absorption intensities caused by benzene ring skeleton of xylenes at 1138 nm, 1670 nm and 2130 nm were negatively correlated with their symmetry, with the order as ortho-xylene < meta-xylene < para-xylene. It illustrates that the higher the chemical structure symmetry, the lower the NIR absorption intensity is.

Moreover, the characteristic bands of some simple group substitutes centered on the benzene were assigned, including ortho-xylene, meta-xylene, para-xylene, phenol, benzyl alcohol, and benzaldehyde. From the results shown in Supplementary Figs S6–S9, it could be found that 1700 nm and 2100–2500 nm were the characteristic bands for NIR modeling of xylenes and 2050–2350 nm could be used of O-H for NIR modeling. These results were meaningful for qualitative and quantitative analysis of isomers by NIR spectroscopy.

### Rapid NIR spectral assignments based on the theory of quantum mechanics

Quantum mechanics theory can construct the molecular model to calculate the theoretical absorption frequency of the molecule for spectral assignment. In order to avoid the complicated experimental process brought by the 2nd spectroscopy and the difference spectrum method, combining density functional theory and the raw NIR spectra shown in Supplementary Fig. S9, we quickly obtained the theoretical fundamental frequency, overtones, combination modes, and vibration groups of the methanol, ethanol, benzene, toluene, ethylbenzene, ortho-xylene, meta-xylene, para-xylene, phenol, benzyl alcohol, and benzaldehyde shown in Table 2.

**Table 2:** The theoretical fundamental frequency, overtones, combination modes, and vibration group of the ramifications of benzene and the characteristic groups.

Compounds	Chemical structure	Characteristic bands (nm)				
		Funda-mental frequency	First double-frequency	Second double-frequency	Third double-frequency	Attribution of characteris-tic bands
Methanol		3199–3338	1649–1964	1111–1391	842–1113	C-H of methyl
		2593	1336–1525	900–1080	682–864	O-H
Ethanol		3348	1726–1970	1163–1395	881–1116	C-H of methylene
		3310	1706–1947	1149–1379	871–1103	C-H of methyl
		3249	1675–1911	1128–1354	855–1083	C-H of methyl and methylene
		3228	1664–1899	1121–1345	850–1076	C-H of methyl and methylene
		3217	1658–1892	1117–1340	847–1072	C-H of methyl
		2604	1342–1531	904–1085	685–868	O-H
Benzene		3122–3163	1609–1860	1084–1318	822–1054	C-H of benzene ring
Toluene		3165–3305	1661–1944	1119–1377	848–1102	C-H of methyl
		3120–3165	1608–1862	1083–1319	821–1055	C-H of benzene ring
Ethyl-benzene		3307	1705–1945	1148–1378	870–1102	C-H of methylene
		3293	1697–1937	1143–1372	867–1098	C-H of methyl
		3268	1685–1922	1135–1362	860–1089	C-H of methylene
		3223	1660–1896	1118–1343	847–1074	C-H of methyl
		3128–3172	1612–1866	1086–1322	823–1057	C-H of benzene ring
Para-xylene		3223–3306	1661–1944	1119–1377	848–1102	C-H of methyl
		3146–3168	1622–1864	1093–1320	828–1056	C-H of benzene ring
Meta-xylene		3227–3253	1677–1957	1130–1386	856–1109	C-H of methyl
		3144–3171	1620–1865	1092–1321	827–1057	C-H of benzene ring
Ortho-xylene		3233–3322	1712–1955	1153–1385	874–1108	C-H of methyl
		3120–3166	1608–1863	1083–1319	821–1055	C-H of benzene ring
Mesitylene		3215–3322	1657–1954	1116–1384	846–1107	C-H of methyl
		3159–3198	1628–1881	1097–1332	831–1066	C-H of benzene ring
Phenol		3122–3164	1609–1861	1084–1318	822–1055	C-H of benzene ring
		2607	1533	1086	869	O -H
Benzyl alcohol		3355	1867–1973	1136–1398	861–1118	C-H of methylene
		3123–3174	1610–1867	1085–1322	835–1058	C-H of benzene ring
		2612	1346–1536	907–1088	687–871	O-H
Benzaldehyde		3479	1793–2046	1208–1450	916–1160	C-H of aldehyde group
		3109–3479	1603–1853	1079–1313	818–1050	C-H of benzene ring
Methyl (CH_3_)		1111–1340, 1649–1892, 1894–2079, 2214–2479				
Methylene (CH_2_)		1111–1340, 1649–1892, 1867–1973, 2214–2341				
Benzene ring (C_6_H_6_)		1117–1176, 1608–1863, 2119–2192				
Aldehyde (CHO)		1229–1294				
Hydroxyl (OH)		1358–1456				

Combined with Supplementary Fig. S9 and Table 2, the attributions of the NIR absorption were easily analyzed and the results were summarized and also listed in Table 2. Amazingly, the NIR absorption peak width of the substance is independent of the number of methyl groups but related to steric hindrance. Moreover, as the increase of the steric hindrance, the absorption peak width of C-H on benzene ring became broader, with the peak width order as ortho-xylene > meta-xylene > para-xylene > benzene. Conversely, in terms of the methyl C-H, the absorption peak shifts to a high wave number as the steric hindrance increases, and the larger the steric hindrance, the narrower the absorption peak is, which is the opposite of the benzene ring. This method provides a guideline for the application of Quantum Mechanics calculation in spectra-structure analysis.

### NIR spectral assignments of natural chemical compounds in CMM for variable selection

**Figure 6:**
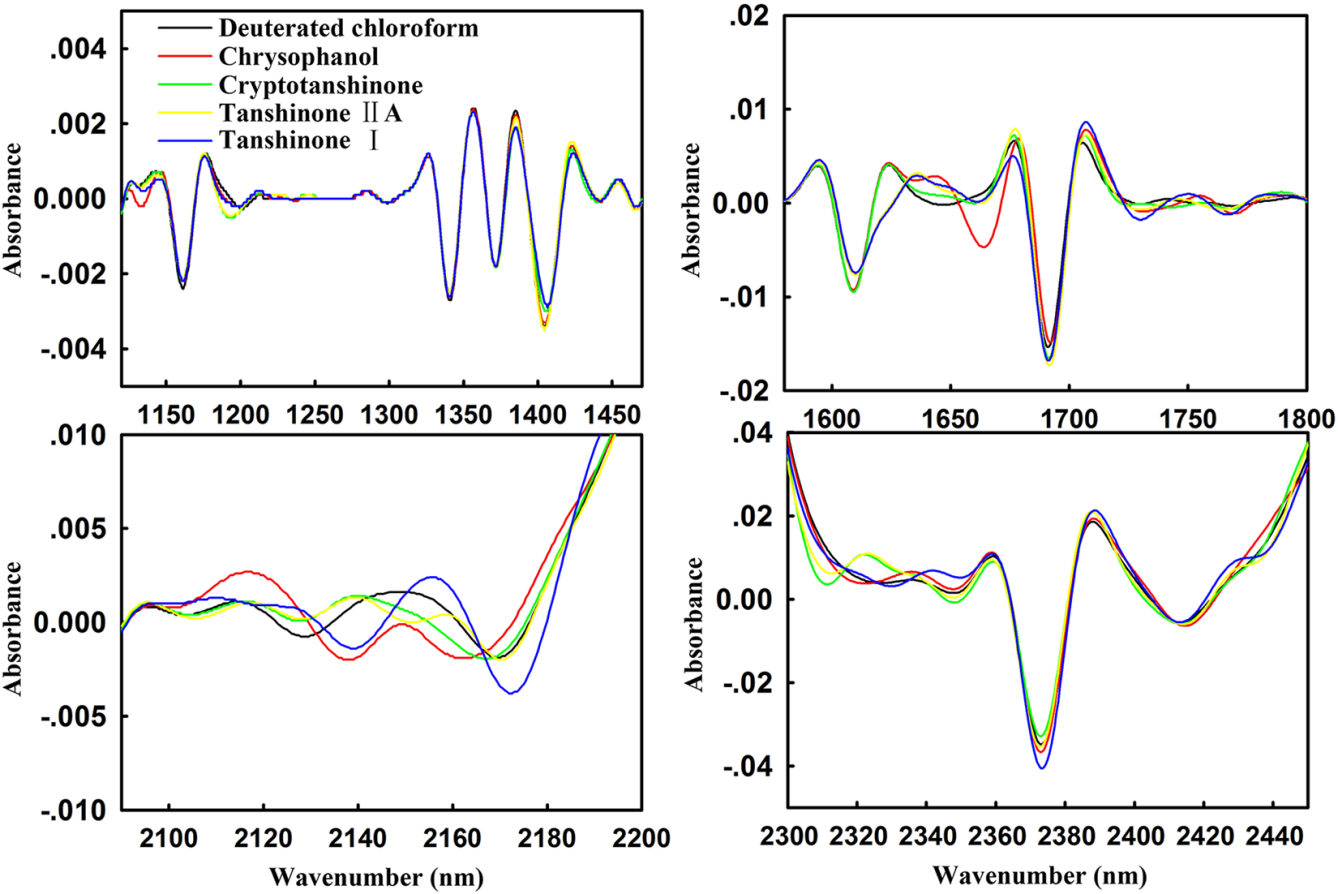
Partial enlargement of the 2nd spectra of anthraquinones. There were significant differences of anthraquinones at 1371–1470, 1620–1838, 1976–2020, and 2094–2200 nm.

As a process analytical technology, NIR is often applied for quality control of CMM production processes. However, due to the complex chemical composition and similar structure of natural chemical compounds in CMM, such as phenylpropanoids, alkaloids, steroids and so on, the original spectral overlap is too serious to analysis. To overcome this problem in some extent, the 2nd spectra of chrysophanol, cryptotanshinone, tanshinone IIA, tanshinone I have been investigated. From Fig. 6, it is clear to observe the significant differences at 1371–1470, 1620–1838, 1976–2020, and 2094–2200 nm. Therefore, 1371–2200 nm could be proposed for NIR modeling as the characteristic band of anthraquinones.

Similarly, the other essential compound groups of natural chemical compounds including phenylpropanoids, alkaloids, moss and so on have been studied. Supplementary Figs S10 to S14 respectively show the full and enlarged view NIR 2nd spectra of simple phenylpropanoids (schizandrin A, schizandrin B, schisandra ester A); Coumarins (psoralen, imperatorin, isopsoralen, isoimperatorin) lignin (honokiol); mosses (cantharidin, menthol, borneol, oleanolic acid, artemisinin, dehydroandrographolide, evodilactone, curcumin dione, curcumol, licorice) acid); alkaloids (fritigenin B, matrine, rutaphanine, oxymatrine, oxalate, isochraticine, reserpine, sinomenine). The characteristic bands of each component category were summarized in Table 3, which were significant for more robust and interpretative models.

**Table 3:** NIR absorption characteristic bands of different natural chemical compounds in CMM.

Compounds	Characteristic bands (nm)
Anthraquinones	1371~1470, 1620~1838, 1976~2020, 2094~2200
Lignins	1160~1280, 1325~1472, 1700~1840, 1970~2200
Coumarins	1370~1480, 1550~1840, 2040~2200, 2301~2470
Simple Phenylalanines	1370~1490, 1117~1285, 1560~1830, 1975~220, 2314~2470
Terpenes	940~1000, 1120~1300, 1380~1480, 1650~1840, 1880~1925, 1970~2180, 2280~2460
Alkaloids	1110~1260, 1370~1495, 1620~1820, 2040~2195, 2300~2460
Flavones	1120~1280, 1350~1550, 1620~1830, 1990~2200, 2300~2460

### NIR spectral assignments for more details of natural chemical compounds in CMM by 2D-COS

**Figure 7:**
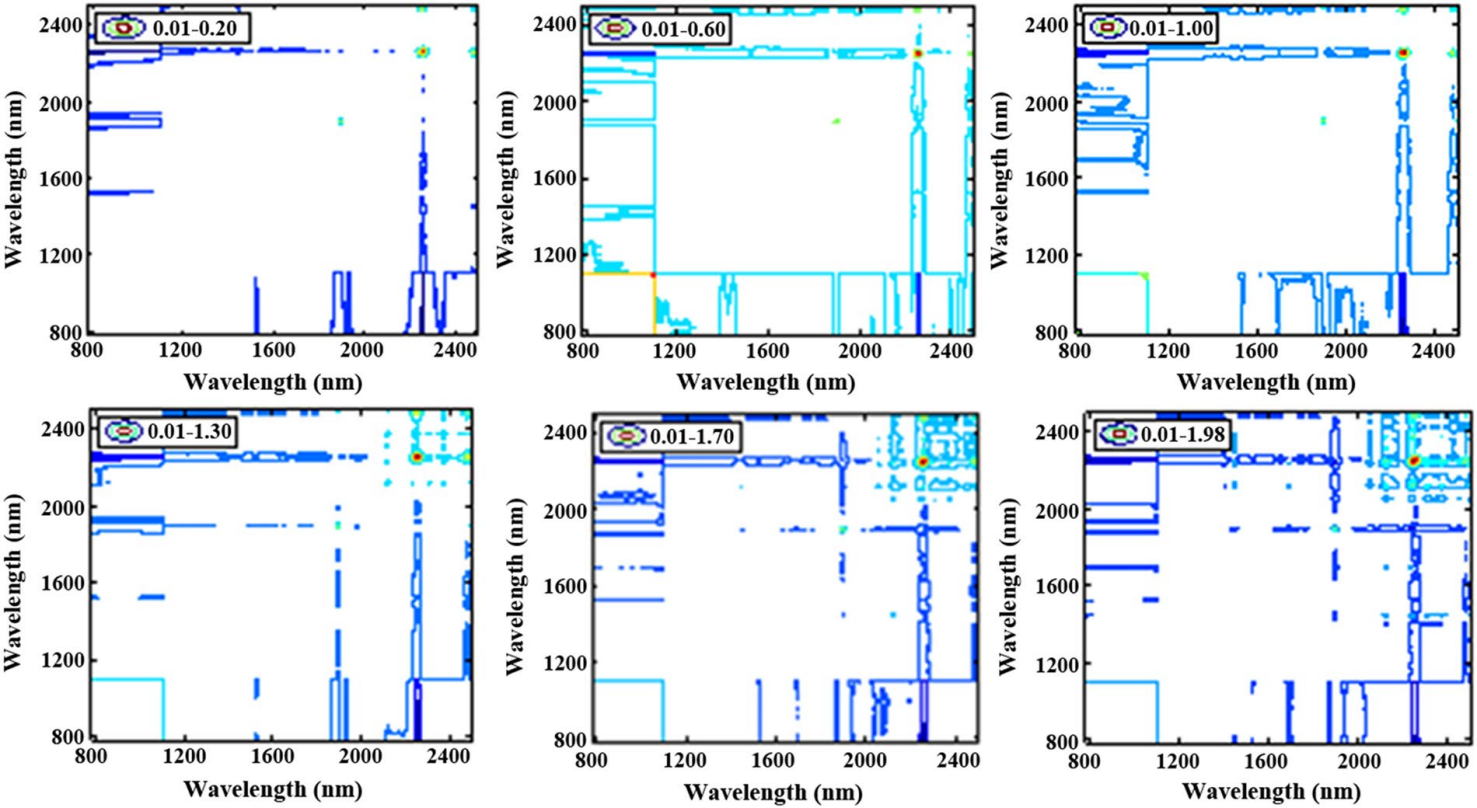
The synchronous 2D-COS spectra of magnolols with different sample concentrations. When the concentration reaches 1.0 mg^−1^, there was an obvious autocorrelation relationship.

The 2nd can assign the characteristic band of a substance, but it is powerless for a more detailed structure analysis. 2D-COS can identify small and weak peaks that are masked in one-dimensional spectrum by subtle spectral changes caused by external disturbances. To determinate the parameter of the two-dimensional correlation analysis, firstly, the 2D-COS of six magnolol solution with gradually increasing concentrations were displayed in Fig. 7. Accidentally, the intensity of autocorrelation peaks is stronger synchronized with the increasing range of sample concentration. Accordingly, the influence of sample concentration was concluded, that is tiny sample concentrations were not suitable for 2D-COS analysis because of unrecognized autocorrelation peaks, and large sample concentrations were not recommended either for increased costs. Secondly, the 2D-COS by three types of concentration interval have been analyzed, and accidentally, we found all had 12 identical autocorrelation peaks (see Supplementary Fig. S15). Interestingly, the number of samples had the same effect on the 2D-COS as the concentration interval (see Supplementary Fig. S16). These provided a meaningful guideline for the 2D-COS analysis of the chemical compound.

**Figure 8:**
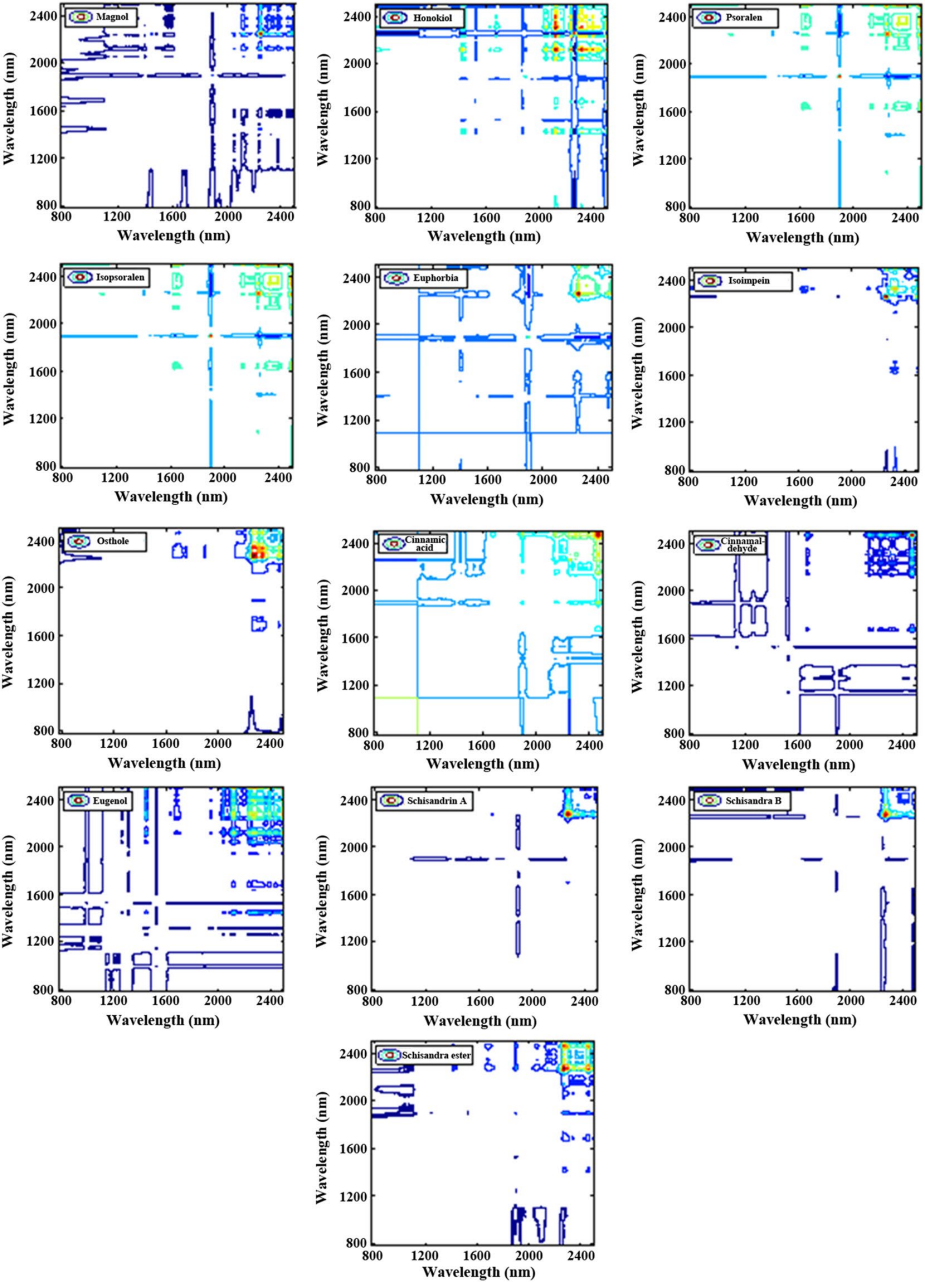
The 2D-COS of 13 kinds of phenylalanines compounds including Magnolol, and honokiol, psoralen, isopsoralen, imperatorin, isoimperatorin, osthole, cinnamic acid, cinnamaldehyde, eugenol, schisandra A, schisandra B, and schisandra ester A.

**Figure 9:**
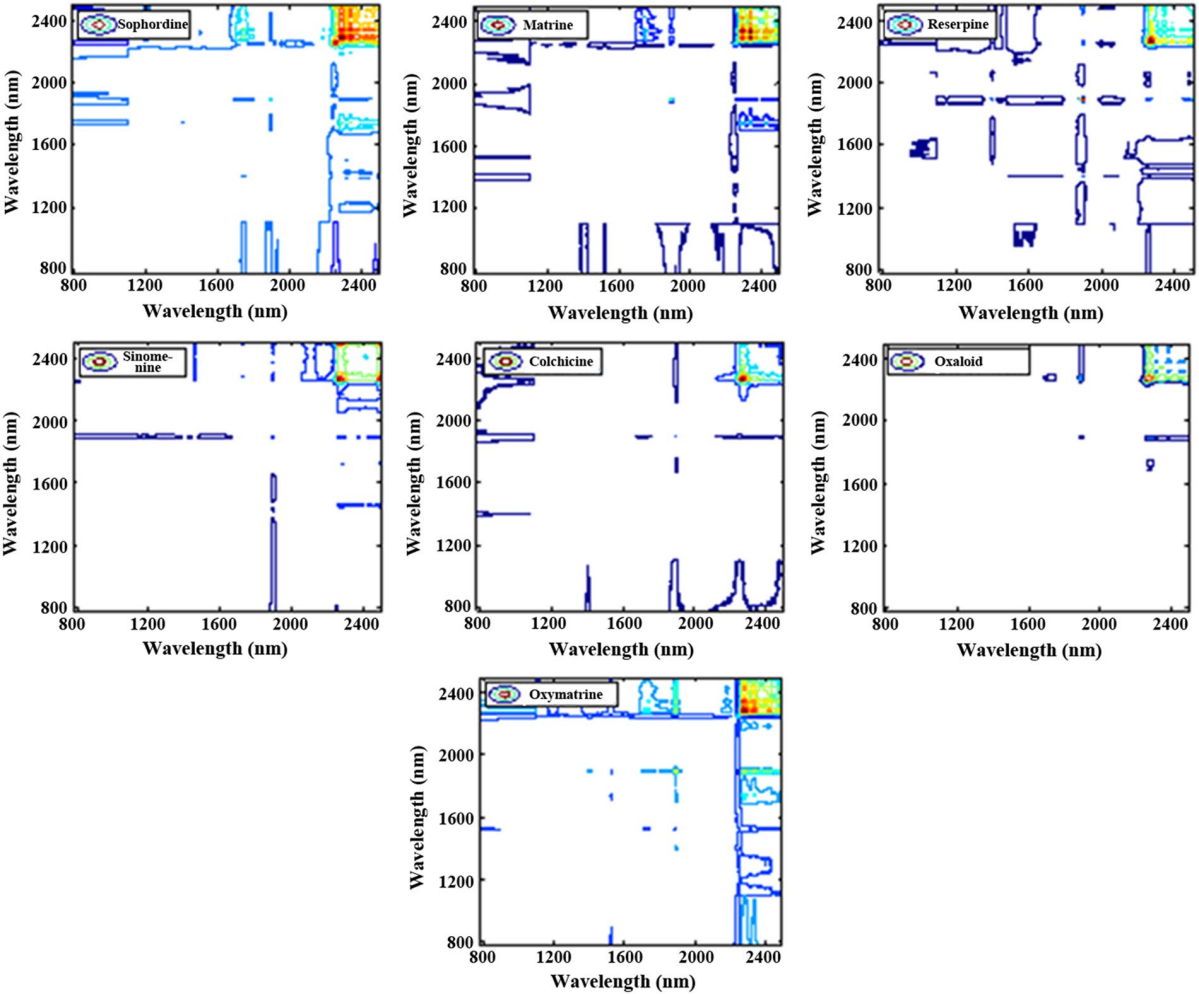
2D-COS of 7 kind of Alkaloids compounds, including alkaloids, matrine, reserpine, sinomenine, colchicine, oxaloid, and oxymatrine.

**Figure 10:**
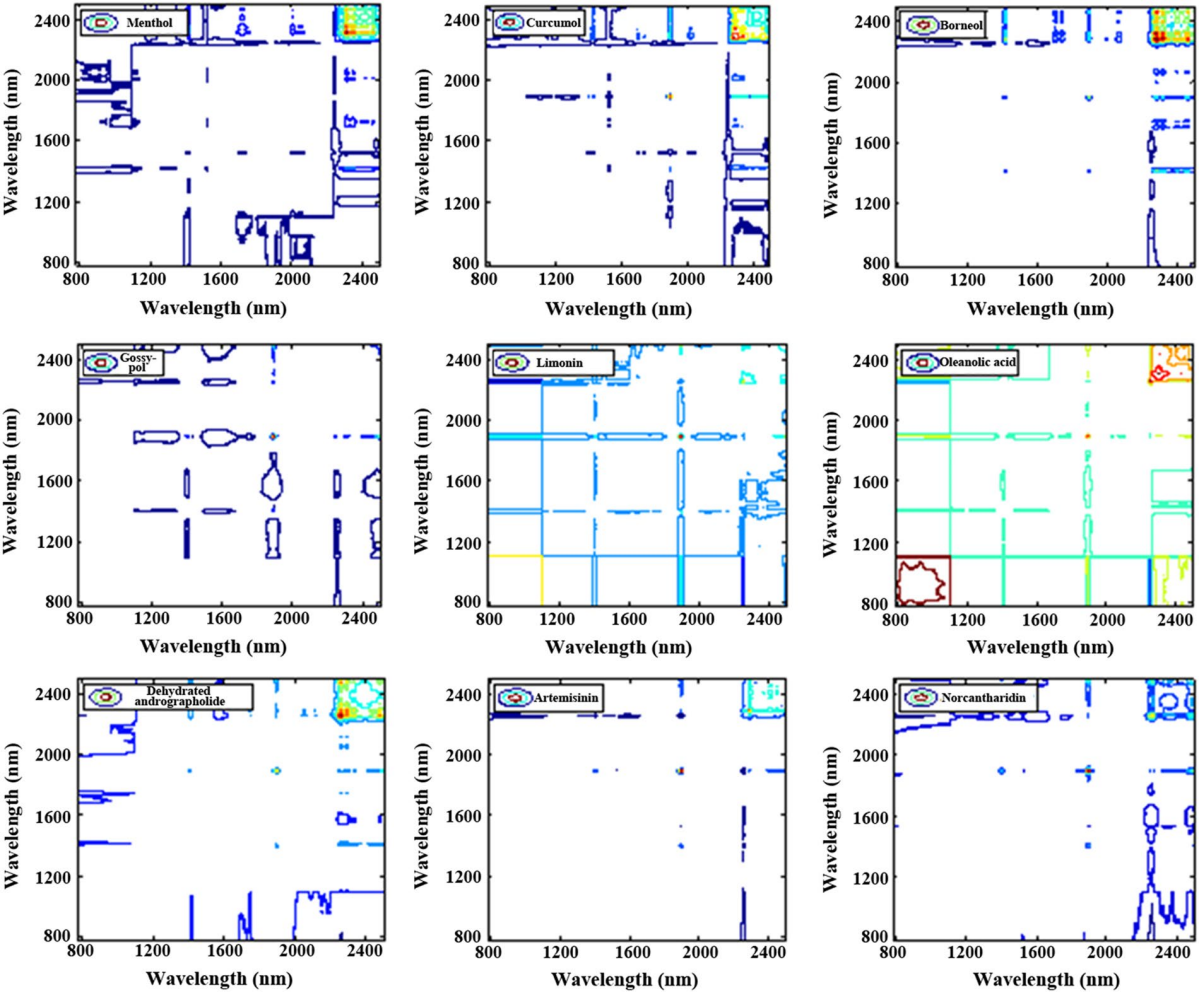
2D-COS of 9 kind of phenylpropanoids compounds, including menthol, curcumol, borneol, gossypol, limonin, oleanolic acid, dehydroandrographolide, artemisinin and norcantharidin.

Owing to the advance of the significant resolution enhancement of heavily overlapped NIR bands, the 2D-COS was proposed to the NIR spectral assignment of CMM. Through analyzing the 2D-COS of 13 phenylpropanoids shown in Fig. 8, the characteristic absorption bands of simple phenylpropanoids (eugenol, cinnamaldehyde, and cinnamic) have been concluded as 1620–1740 nm, 1863–1963 nm, and 2200–2500 nm, and those of Lignins as 1387–1468 nm, 1619–1714 nm, and 2200–2500 nm, Coumarins as 1387–1468 nm, 1619–1714 nm, and 2200–2500 nm. By the same method, the 2D-COS of 7 alkaloid compounds and 9 terpenoid compounds were displayed in Figs 9 and 10. Obviously, the small and weak peaks hidden in one-dimensional spectra can be observed easily.

The NIR absorbance is dominated by the functional groups containing hydrogen atoms (e.g. OH, CH, NH). Accordingly, further spectra-structure research of these 29 compounds were performed, and the details information of which were concluded in Tables 4, 5 and 6, respectively. These indicated that 2D-COS could be seen as an excellent method for the NIR spectral assignment of complex natural chemical compounds.

**Table 4:** The characteristic absorption band and structure of 13 phenylpropanoids compounds.

Category	Chemical compound	Chemical structure	NIR absorption groups	Characteristic absorption bands (nm)	Attribution of characteristic bands	Common characteristic absorption bands (nm)
Simple phenylalanine	Eugenol		C-H in benzene ring, OCH_3_, CH_3_, CH_2_, and C=C; O-H in benzene ring	1419–1472	The combination absorption bands of C-H in CH_2_ and O-H in benzene ring	1620–1740 1863–1963 2000–2500
				1617–1782	The combination absorption bands of C-H in C=C, benzene ring, and OCH_3_	
				1920–1959		
				1992–2500	The characteristic absorption bands of organic nitrogen compound	
	Cinnamaldehyde		C-H in CHO, C=C and benzene ring	1643–1725	The absorption bands of C-H in benzene ring	
				2095–2373	The absorption bands of C-H in CHO	
				2415–2500	The absorption bands of C-H in C=C	
	Cinnamic acid		O-H in carboxylic group; C-H in C=C and benzene ring	1649–1703	The absorption bands of C-H in benzene ring	
				1865–1922	The combination absorption bands of C=O and O-H in carboxylic group	
				2073–2500	The combination absorption bands of deuterium chloroform solvent and C-H and C-C in benzene ring	
Lignin	Magnolol and Honokiol		C-H in benzene ring, CH_2_, and C=C; O-H in benzene ring	1383–1472	The combination absorption bands of C-H in CH_2_ and O-H in benzene ring	1387–1468 1619–1714 1873–1912 2018–2180 2215–2500
				1615–1739	The combination absorption bands of C-H in benzene ring and C=C	
				1864–1969		
				2018–2500		
	Schizandrin A		C-H in benzene ring, OCH_3_, CH_3_, and CH_2_	1391–1412	The combination absorption bands of C-H in CH_2_ and CH_3_	
				1640–1800	The absorption bands of C-H in OCH_3_	
				1870–1915		
				2200–2500	The combination absorption bands of deuterium chloroform solvent and C-H and C-C in benzene ring	
	Schizandrin B		C-H in benzene ring, OCH_3_, CH_3_, OCH_2_, and CH_2_	1385–1421	The combination absorption bands of C-H in CH_2_ and CH_3_	
				1649–1800	The absorption bands of C-H in OCH_3_	
				1864–1910		
				2204–2500	The combination absorption bands of deuterium chloroform solvent and C-H and C-C in benzene ring	
	Schisandra ester A		C-H; O-H; and C=O in ester groups	1376–1452		
				1644–1768	The combination absorption bands of C-H in benzene ring and OCH_3_	
				1874–1926		
				2035–2079		
				2115–2177	The combination absorption bands of deuterium chloroform solvent and C-H and C-C in benzene ring	
				2235–2500		
Coumarin	Psoralen and Isopsoralen		C-H in benzene ring and C=C	1381–1422	The absorption bands of C-H in benzene ring	1379–1415 1595–1785 1862–1920 2046–2500
				1590–1695	The combination absorption bands of C-H in benzene ring and C=C with oxygen atom	
				1863–1922		
				2058–2500		
	Imperatorin and Isoimperatorin		C-H in benzene ring, CH_3_, CH_2_, and C=C	1388–1413	The absorption bands of C-H in benzene ring	
				1594–1677	The combination absorption bands of C-H in benzene ring and C=C with ester groups	
				1864–1914		
				2057–2500		
	Osthole		C-H in benzene ring, OCH_3_, CH_3_, CH_2_, and C=C	1328–1429	The combination absorption bands of C-H in CH_2_ and CH_3_	
				1609–1802	The combination absorption bands of C-H in C=C, CH_3_, and OCH_3_	
				1870–1927		
				2041–2500

**Table 5:** The characteristic absorption bands and structure assignment of 7 alkaloid compounds.

Chemical compound	Chemical structure	NIR absorption groups	Characteristic absorption bands (nm)	Attribution of characteristic bands
Sophordine and Matrine		C-H and N-H	1386–1451	The absorption bands of C-H in CH_2_
			1688–1800	The combination absorption bands by symmetric and antisymmetric oscillations of C-H in CH_2_
			1819–1913	
			2165–2500	The characteristic absorption bands of organic nitrogen compound
Reserpine		C-H	1385–1416	The absorption bands of C-H in CH_2_
			1863–1919	
			2113–2500	The combination absorption bands of organic nitrogen compound and C-H in benzene ring
Sinomenine		C-H, O-H, and N-H	1386–1480	The combination absorption bands of C-H in CH_2_, N-H and C-H in benzene ring
			1632–1803	The combination absorption bands of C-H in CH_2_ and CH_3_
			1867–1915	
			1936–2150	The absorption bands of N-H
			2219–2500	The combination absorption bands of organic nitrogen compound and C-H in benzene ring
Colchicine		C-H	1339–1506	The combination absorption bands of C-H in CH_2_ and N-H in CONH_2_
			1611–1800	The combination absorption bands of C-H in CH_2_ and CH_3_
			1874–1915	
			2213–2500	The combination absorption bands of organic nitrogen compound and C-H in benzene ring
Oxysophori-dine and Oxymatrin		C-H and N-H	1380–1421	The absorption bands of C-H in CH_2_
			1656–1759	The combination absorption bands of C-H in CH_2_ and C=C
			1862–1930	
			2149–2500	The characteristic absorption bands of organic nitrogen compound

**Table 6:** The characteristic absorption band and structure assignment of 9 terpenoid compounds.

Chemical compound	Chemical structure	NIR absorption groups	Characteristic absorption bands (nm)	Attribution of characteristic bands
Menthol		O-H, C-H of CH_3_, CH_2_, and RCH(CH_3_)_2_	1395–1435	The combination absorption bands of C-H in RCH(CH_3_)_2_ and O-H in alcoholic hydroxyl
			1683–1781	The combination absorption bands by symmetric and antisymmetric oscillations of C-H in CH_2_
			1870–1909	The combination absorption bands of O-H in alcoholic hydroxyl
			1984–2081	The combination absorption bands of C-H in CH_2_ and CH and O-H in alcoholic hydroxyl
Borneol		O-H, C-H of CH_3_ and CH_2_	1386–1432	The combination absorption bands of C-H in RCH(CH_3_)_2_ and O-H in alcoholic hydroxyl
			1671–1782	The combination absorption bands of C-H in CH_3_ and CH_2_
			1869–1919	
			1978–2009	The combination absorption bands by stretching vibration and bending vibration of O-H
			2030–2089	The absorption bands by deformation vibration of O-H
			2223–2500	The combination absorption bands of C-H in CH_2_ and O-H in alcoholic hydroxyl
Curcumol		O-H, C-H of CH_3_, C=C, and CH_2_	1386–1440	The absorption bands of C-H in RCH(CH_3_)_2_
			1626–1649	The absorption bands of C-H in C=C
			1674–1778	The combination absorption bands of C-H in CH_2_ and CH_3_
			1867–1925	
			1993–2059	The combination absorption bands by stretching vibration and bending vibration of O-H
			2102–2132	
			2206–2500	The combination absorption bands of C-H in CH_2_ and O-H in alcoholic hydroxyl
Gossypol		C=O, O-H, C-H of CH_3_, CH_2_, and benzene ring	1381–1482	The combination absorption bands of C-H in CH_2_ and CH_3_ and O-H in benzene ring
			1866–1921	
			2059–2500	The combination absorption bands of C-H and O-H in benzene ring
Oleanolic acid		C=O, O-H, C-H of CH_3_, CH_2_ and C=C	1395–1426	The combination absorption bands of C-H in CH_2_
			1677–1780	The combination absorption bands of C-H in CH_3_
			1860–1919	The absorption bands by stretching vibration of uncombined O-H
			2222–2500	The combination absorption bands of O-H in alcoholic hydroxyl and C-H in CH_2_ and CH_3_
Limonin		C-H of CH_3_, C=C, and CH_2_	1387–1414	The combination absorption bands of C-H in CH_3_ and CH_2_
			1869–1919	The combination absorption bands of C=O and C-H in OCH_3_
			2217–2500	The overlapping absorption of C-H in CH_2_
		C=O, O-H, C-H of CH_3_ and C=C	1395–1426	The combination absorption bands of O-H in alcoholic hydroxyl and C-H in CH_2_
			1677–1780	
			1860–1919	The combination absorption bands of O-H in alcoholic hydroxyl and C=O
			2222–2500	The combination absorption bands of O-H in alcoholic hydroxyl and C-H in CH_2_ and CH_3_
Artemisinin		O-H, C-H of CH_3_ and CH_2_	1384–1422	The combination absorption bands of C-H in CH_3_ and CH_2_
			1665–1779	The absorption band by stretching vibration of C-H in CH_2_
			1866–1936	
			2222–2500	The combination absorption bands of O-H in alcoholic hydroxyl and C-H in CH_2_ and CH_3_
Nor cantharidin		C=O, C-H of CH_3_, CH_2_, and CH	1386–1418	The absorption bands by stretching vibration of C-H in CH_2_
			1668–1734	The absorption bands by stretching vibration of C-H in CH_3_
			1865–1919	The absorption bands by stretching vibration of C=O
			2218–2500	The combination absorption bands of C-H in CH_3_, CH_2_, and CH
Dehydroan-drographolide		C=O, O-H, C-H of CH_3_, CH_2_ and C=C	1382–1431	The combination absorption bands of O-H in alcoholic hydroxyl and C-H in CH_2_
			1673–1761	The absorption bands by stretching vibration of C-H in CH_2_
			1859–1912	The absorption bands by stretching vibration of C=O
			1986–2171	The absorption bands by stretching vibration of uncombined O-H
			2210–2500	The combination absorption bands of O-H in alcoholic hydroxyl and C-H in CH_2_ and CH_3_

Supplementary Table S2 showed a comparison of the analytical results of the second derivative and 2D-COS. It can be seen that the characteristic bands of several kinds of compounds assigned by these two methods were very close. For example, the characteristic bands of terpenoids assigned by the second derivative were “940–1000, 1120–1300, 1380–1480, 1650–1840, 1880–1925, 1970–2180, 2280–2460 (nm)”, while one assigned by 2D-COS were “388–1442, 1676–1761, 1864–1920, 1990–2145, 2320–2500 (nm)”. Almost all of the characteristic variables obtained by 2D-COS were within the range of the bands assigned by the second derivative spectroscopy method, which verified that the 2D-COS was more accurate.

## Discussion

According to the spectra-structure interrelationship, a serial of critical results of NIR absorption bands have been revealed progressively. Firstly, for skeleton structure, sp^2^ hybridization can induce a higher absorption frequency than sp^3^ hybridization. As to the influence of substituent, with the increase of the number of electrons donating substituent, absorption intensity of methyl substituted benzene increases proportionally, and the displacement distance of absorption peak became larger. In addition, as another key factor, the temporal steric could decrease the NIR absorption intensity regularly. Furthermore, not only chemical structure with specific property, but also complex natural chemical compounds, both can be assigned for more interpretative NIR model. 2D-COS was more accurate for characteristic bands and detail information. These laid a solid theoretical foundation of qualitative and quantitative analysis for more interpretative model based on spectral assignment.

## Methods

### Experimental instruments and materials

Carbon tetrachloride, cyclohexane, benzene, toluene, o-xylene, m-xylene and p-xylene, mesitylene, and phenol were purchased from Beijing Chemical Reagent Factory. Ethylbenzene, benzyl alcohol and benzaldehyde were purchased from Tianjin Chemical Reagent Factory. Deuterated chloroform was purchased from Cambridge Isotope Laboratories, Inc. (Massachusetts, USA). All reference standards were supplied by the Chinese Food and Drug Inspection Institute (Beijing, China).

### Sample preparation of NIR raw spectra

2.2 mL of benzene, 2.6 mL of toluene, 3.0 mL of ethylbenzene, 3.1 mL of o-xylene, 3.1 mL of m-xylene, 3.1 mL of p-xylene, 3.5 mL of mesitylene, 2.5 mL of benzaldehyde, 2.6 mL of benzyl alcohol, and 2.4 g of phenol were measured precisely, and diluted with carbon tetrachloride to obtain 1 mol/L stock solution.

Furthermore, 4, 8, 12, 192, 196, 200 μL stock solution of benzene, toluene, xylenes and mesitylene were diluted respectively with carbon tetrachloride to obtain a series of solution with volume fraction from 0.2% to 10%. Similarly, 12–200 μL stock solution of methanol and ethanol were diluted respectively with carbon tetrachloride to obtain a series of solution with volume fraction from 0.6% to 10%.

### The collection of NIR raw spectra, 2nd spectra and difference spectra collection

The spectra were collected by FOSS RLA holographic grating NIR spectrometer (Metrohm China, China). Spectra collection was set as transmission mode. The background was set as air inside the instrument. The average spectrum was gained by three parallel spectra collected with the resolution as 0.5 nm, scanning range as 400–2500 nm, and scanning times as 32.

The NIR difference spectra of toluene, ethylbenzene, o-xylene, m-xylene, p-xylene, mesitylene, phenol, benzyl alcohol, and benzaldehyde were respectively obtained by respective mutual subtraction of the raw spectra.

### NIR 2D-COS collection

The samples were prepared according to Supplementary Table S1. The NIR raw spectra were collected by the same method above. Two-dimensional correlation analysis was carried out on the raw spectra of each substance disturbed by concentration. According to the peak position and number of autocorrelation peaks in the synchronization spectra, the NIR absorption characteristics of these substances were attributed.

### Software

Data was collected by VISION spectra collection and processed by analysis software (Metrohm China, China). Data pre-processing was conducted by Unscrambler 9.7 software (CAMO software co, Norway). Data of 2D-COS was pre-processed by self-programming of MATLAB (The MathWorks Co., American).

## Supplementary information

Supplementary Information
