# Supplementary material for: Systematic discovery about NIR spectral assignment from chemical structural property to natural chemical compounds
Source: Sci Rep. 2019 Jul 1;9:9503. doi: 10.1038/s41598-019-45945-y (PMC6603013; doi:10.1038/s41598-019-45945-y)
Supplement: Supplementary file 1 — Supplementary Information [file 41598_2019_45945_MOESM1_ESM.pdf]

Systematic discovery about NIR spectral assignment from chemical structural property to natural chemical compounds

Lijuan Ma[1, 2, 3#], Yanfang Peng[4#], Yanling Pei[1, 2, 3], Jingqi Zeng[5], Haoran Shen[1, 2, 3], Junjie Cao[1, 2, 3], Yanjiang Qiao[1, 2, 3*], and Zhisheng Wu[1, 2, 3*]

Legends for supplementary figures and tables

Figure S1 The NIR raw spectrum of benzene and cyclohexane. There are obvious differences between the benzene and cyclohexane at 890 nm, 1210 nm, 1400 nm, and 1760 nm.

Figure S2 The partially enlarged DS of methyl substituted benzenes and benzene. At 1138 nm, 1670 nm, and 2130 nm, the order of absorption peak intensity from large to small was ethylbenzene > toluene > xylene > mesitylene.

Figure S3 The NIR DS between methyl substituted benzenes and toluene. From this figure, the differences at 2330 nm caused by methyl could be seen and the order of absorption peak intensity from large to small was ethylbenzene > toluene > xylene > mesitylene.

Figure S4 The partially enlarged view of the NIRS DS between xylenes and benzene. the absorption intensities caused by benzene ring skeleton of xylenes at 1138 nm, 1670 nm and 2130 nm were negatively correlated with their symmetry, with the order as ortho-xylene < meta-xylene < para-xylene.

Figure S5 The partially enlarged view of NIRS DS between xylenes and benzene and NIRS DS between xylenes and toluene.

Figure S6 The two subtraction DS of xylenes. There were some certain absorptive distinctions of ortho-xylene, meta-xylene, and para-xylene at 1700 nm and combination mode at 2100-2500 nm.

Figure S7 The NIR 2nd spectra of benzene, phenol, benzyl alcohol, benzaldehyde. In this figure, phenol and benzyl alcohol both had absorption at 970 nm and 1410 nm, while benzaldehyde did not.

Figure S8 The NIR DS of phenol, benzyl alcohol, benzaldehyde, and benzene, in which phenol and benzyl alcohol had strong characteristic absorption at 1410 nm and there were obvious distinctions at the range of 2050-2350 nm.

Figure S9 The raw NIR spectra of the methanol, ethanol, benzene, toluene, ethylbenzene, ortho-xylene, meta-xylene, para-xylene, phenol, benzyl alcohol, and benzaldehyde.

Figure S10 The partial enlargement of the 2nd spectra of phenylalanines.

Figure S11 The partial enlargement of the 2nd spectra of coumarins, including imperatorin, isoimperatorin, psoralen, isopsoralen, and osthole.

Figure S12 The partial enlargement of the 2nd spectra of lignins, including Schisandra B, Schisandra A, Schisandrin A, Schizandrol, and Ampicin.

Figure S13 The partial enlargement of the 2nd spectra of alkaloids, including fritillaria B, matrine, evodiamine, oxymatrine, oxidized saponin, isorubicin, reserpine, and sinomenine.

Figure S14 The partial enlargement of the 2nd spectra of terpenoids, including cantharidin, menthol, borneol, artemisinin, oleanolic acid, dehydroandrographolide, eucalyptus lactone, zedoary diketone, curcumol, and glycyrrhetinic acid.

Figure S15 The synchronous 2D-COS of magnolols with different sample concentration intervals. There are 12 same autocorrelation peaks caused by this 3 kinds of sample concentration intervals at 1339 nm, 1444 nm, 1631 nm, 1690 nm,

Figure S16 The synchronous 2D-COS of magnolols with different sample sizes. There are 12 same autocorrelation peaks caused by these 3 kinds of sample sizes at 1339 nm, 1444 nm, 1631 nm, 1690 nm, 1946 nm, 2049 nm, 2120 nm, 2286 nm, 2307 nm, 2343 nm, 2372 nm, and 2349 nm.

Table S1 The sample preparation for 2D-COS.

Table S2 The characteristic bands of several kinds of compounds assigned by the second derivative and 2D-COS, respectively.

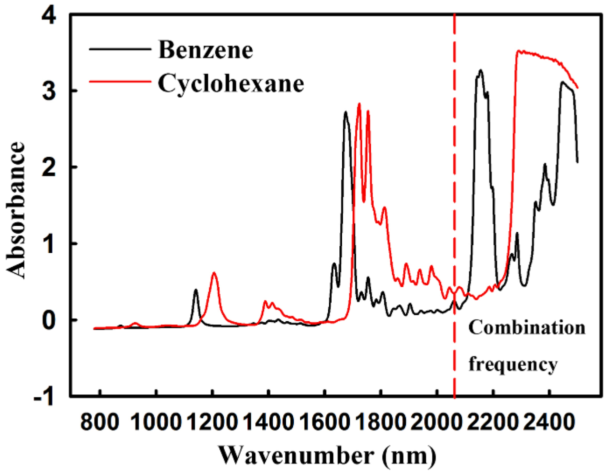

Figure S1 The NIR raw spectrum of benzene and cyclohexane. There are obvious differences between the benzene and cyclohexane at 890 nm, 1210 nm, 1400 nm, and 1760 nm.

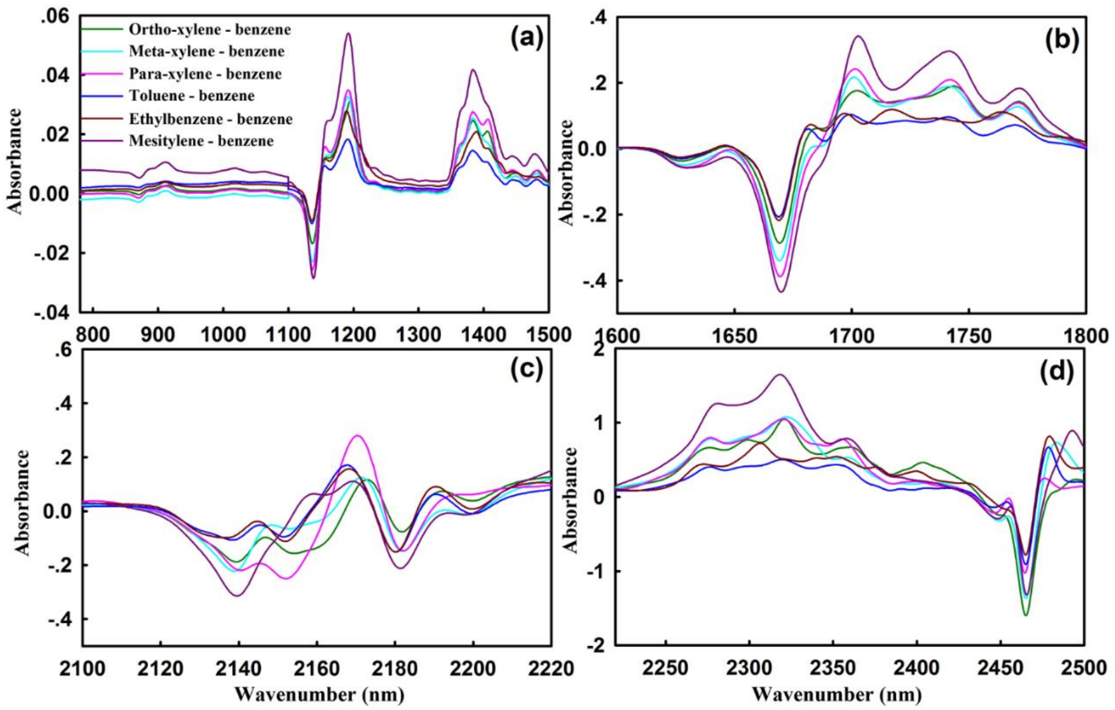

Figure S2 The partially enlarged DS of methyl substituted benzenes and benzene. At 1138 nm, 1670 nm, and 2130 nm, the order of absorption peak intensity from large to small was ethylbenzene > toluene > xylene > mesitylene.

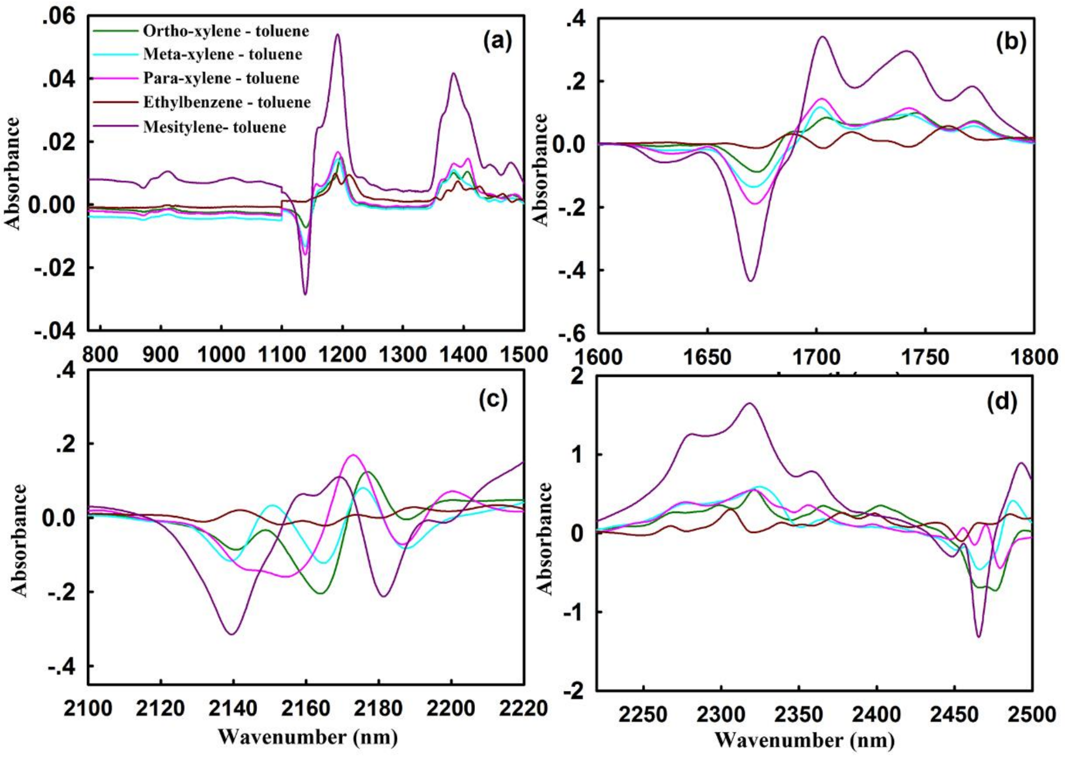

Figure S3 The NIR DS between methyl substituted benzenes and toluene. From this figure, the differences at 2330 nm caused by methyl could be seen and the order of absorption peak intensity from large to small was ethylbenzene > toluene > xylene > mesitylene.

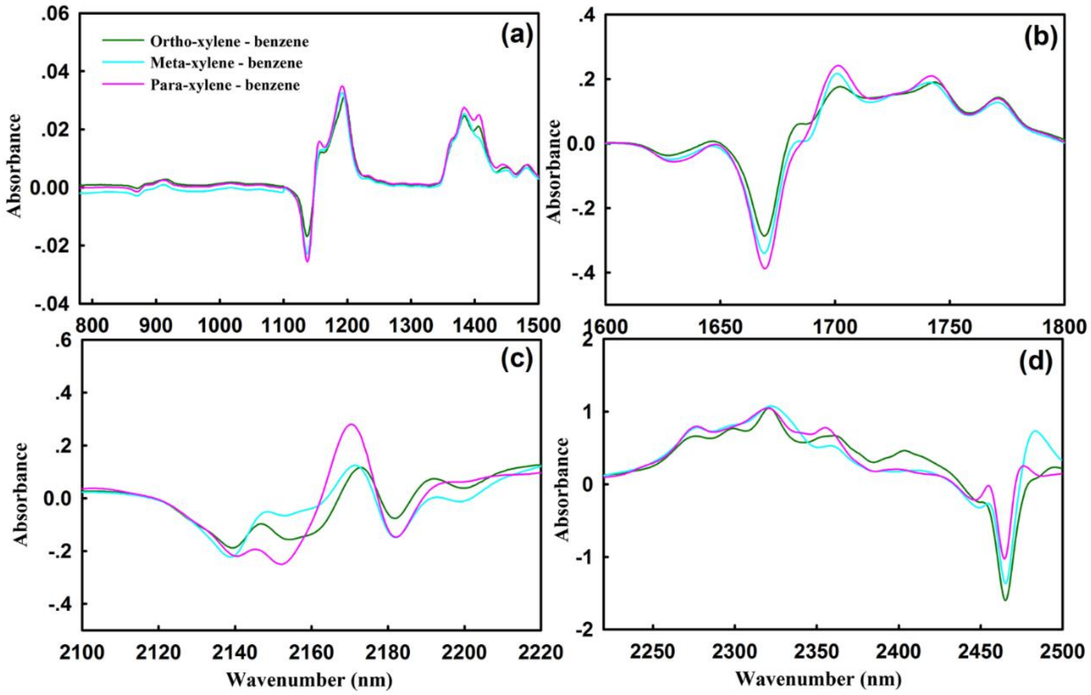

Figure S4 The partially enlarged view of the NIRS DS between xylenes and benzene. the absorption intensities caused by benzene ring skeleton of xylenes at 1138 nm, 1670 nm and 2130 nm were negatively correlated with their symmetry, with the order as ortho-xylene < meta-xylene < para-xylene.

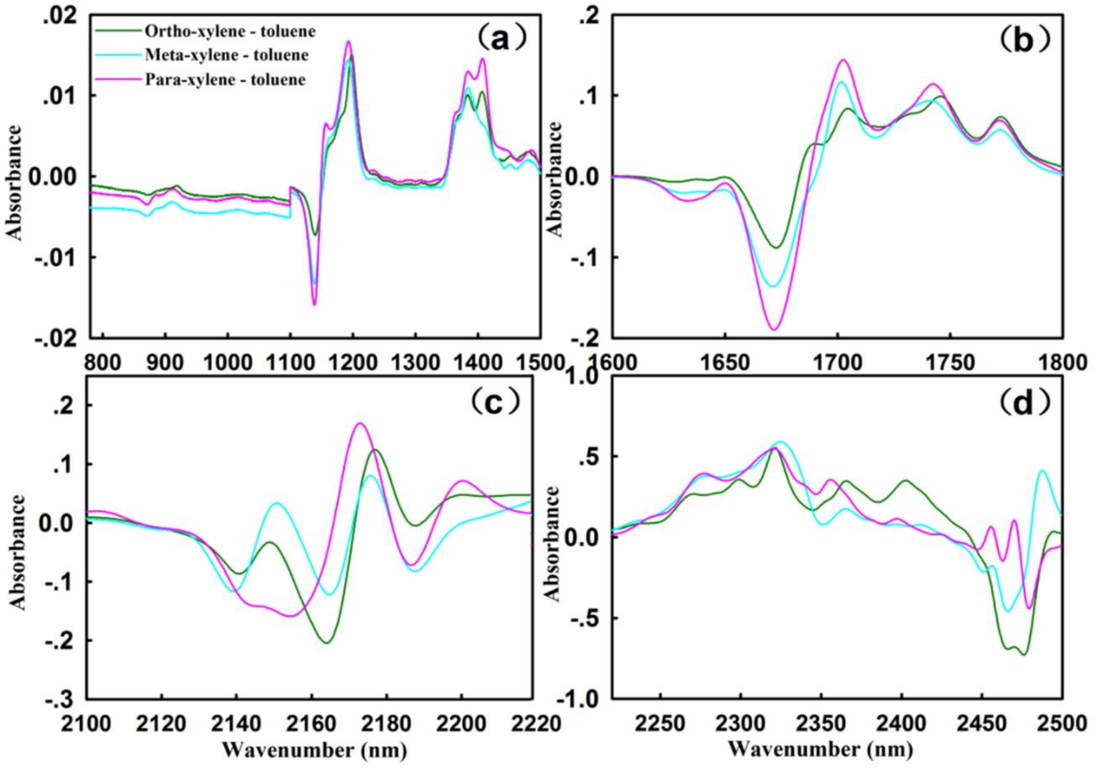

Figure S5 The partially enlarged view of NIRS DS between xylenes and benzene and NIRS DS between xylenes and toluene.

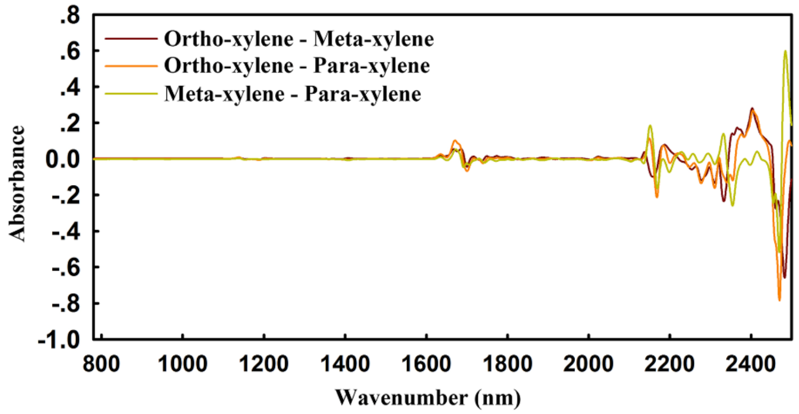

Figure S6 The two subtraction DS of xylenes. There were some certain absorptive distinctions of ortho-xylene, meta-xylene, and para-xylene at 1700 nm and combination mode at 2100-2500 nm.

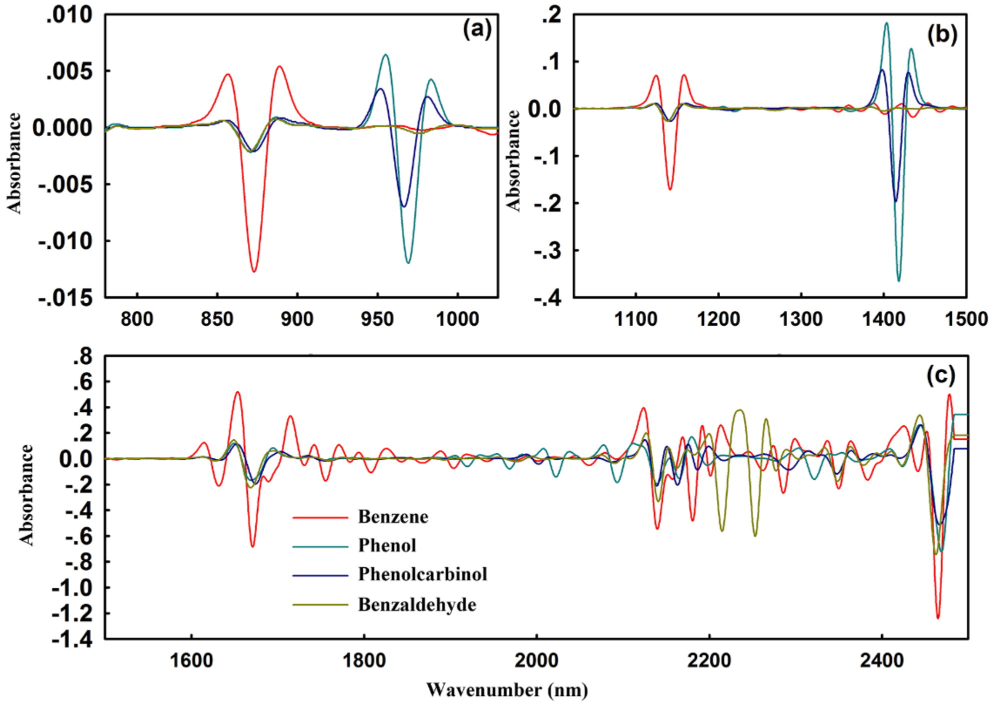

Figure S7 The NIR 2nd spectra of benzene, phenol, benzyl alcohol, benzaldehyde. In this figure, phenol and benzyl alcohol both had absorption at 970 nm and 1410 nm, while benzaldehyde did not.

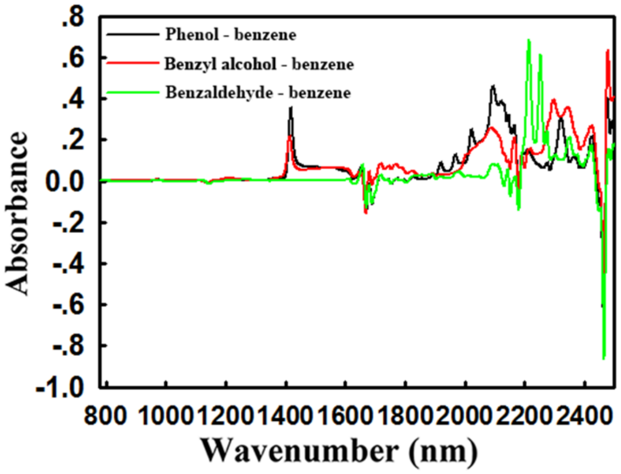

Figure S8 The NIR DS of phenol, benzyl alcohol, benzaldehyde, and benzene, in which phenol and benzyl alcohol had strong characteristic absorption at 1410 nm and there were obvious distinctions at the range of 2050-2350 nm.

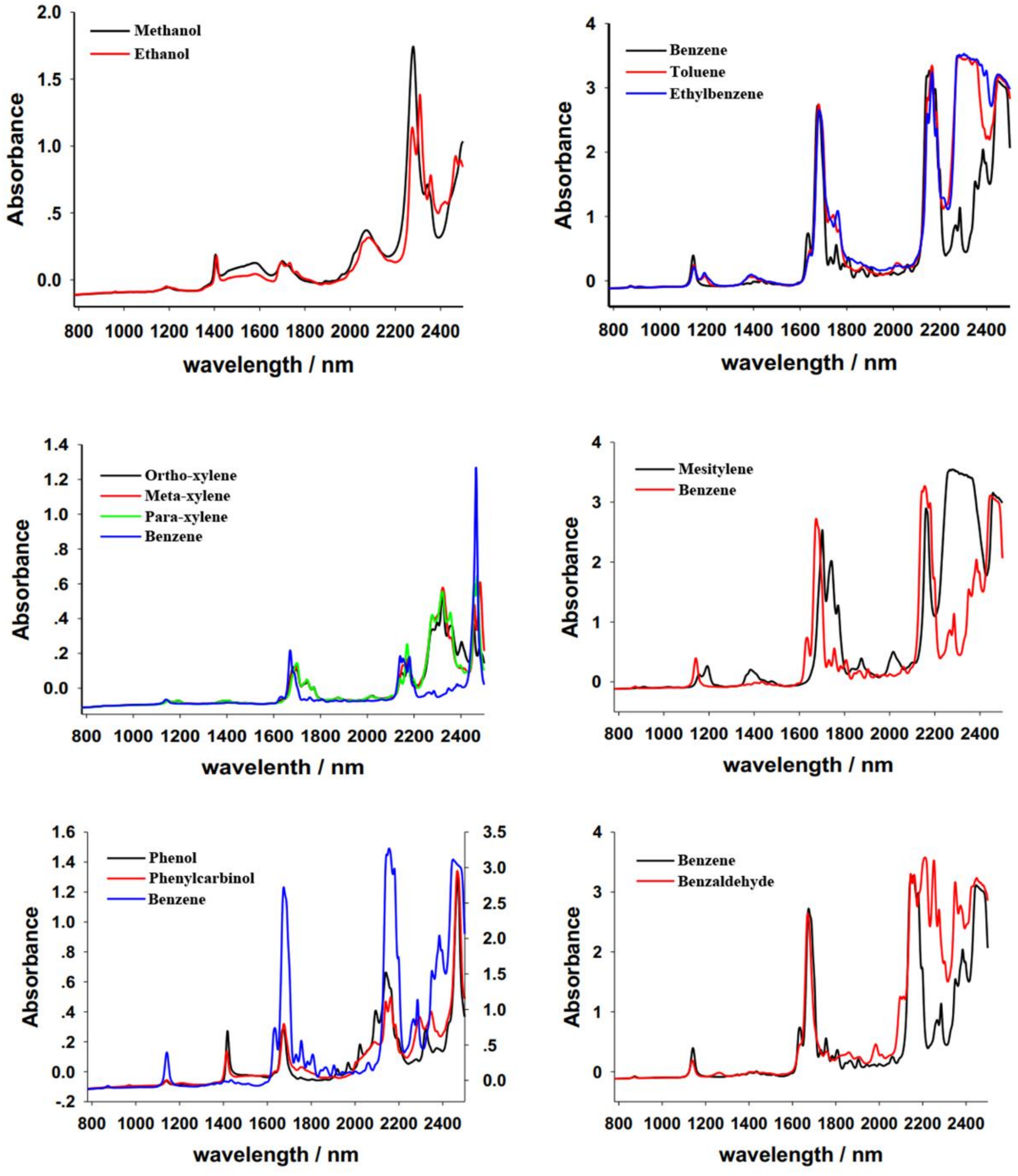

Figure S9 The raw NIR spectra of the methanol, ethanol, benzene, toluene, ethylbenzene, ortho-xylene, meta-xylene, para-xylene, phenol, benzyl alcohol, and benzaldehyde.

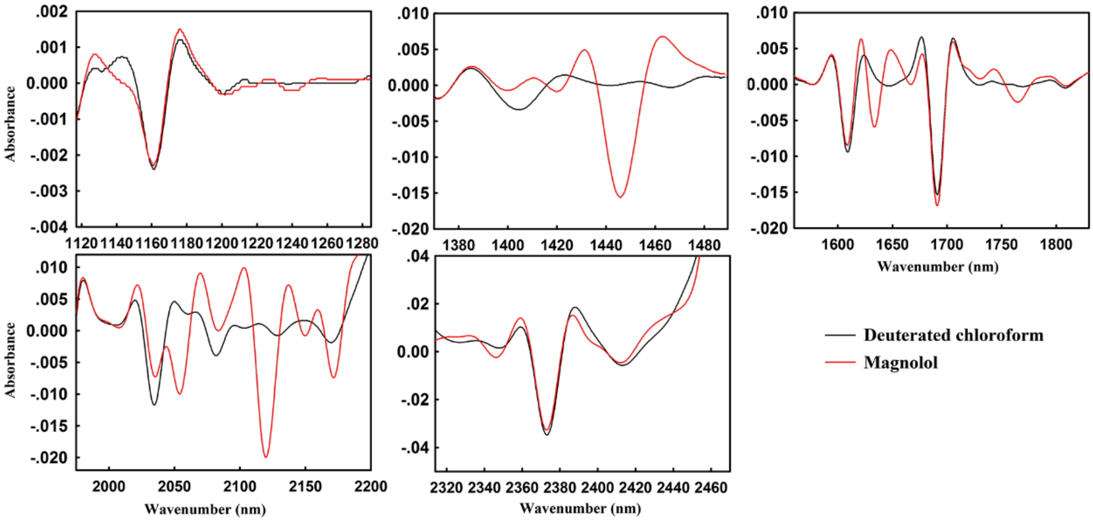

Figure S10 The partial enlargement of the 2nd spectra of phenylalanines.

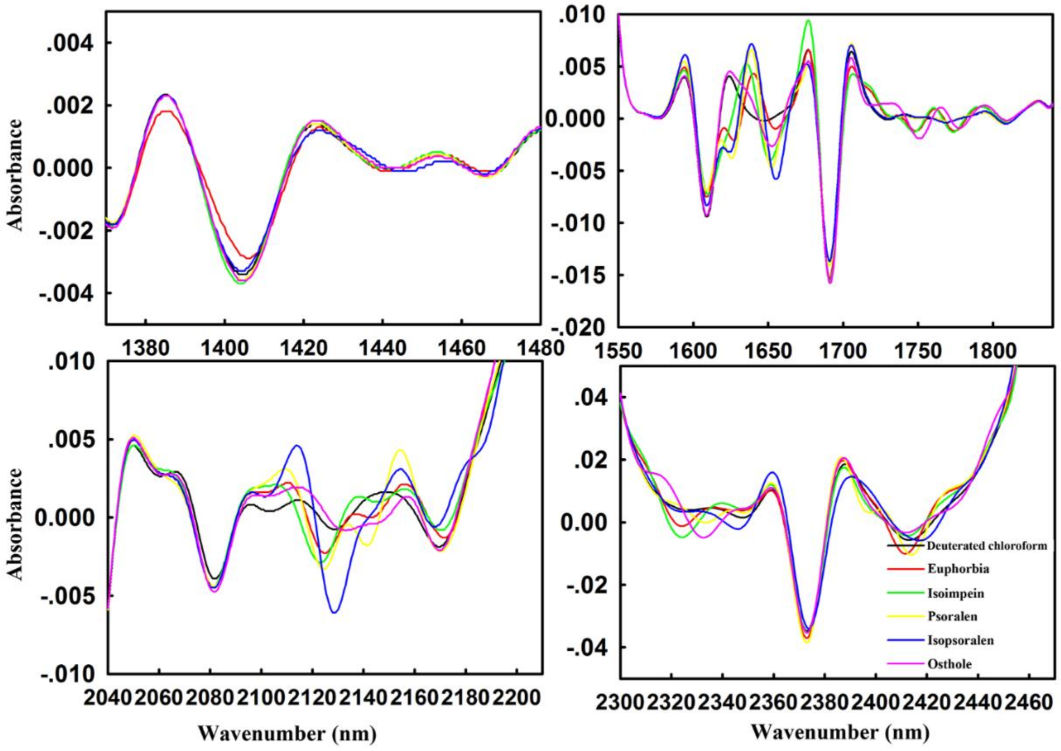

Figure S11 The partial enlargement of the 2nd spectra of coumarins, including imperatorin, isoimperatorin, psoralen, isopsoralen, and osthole.

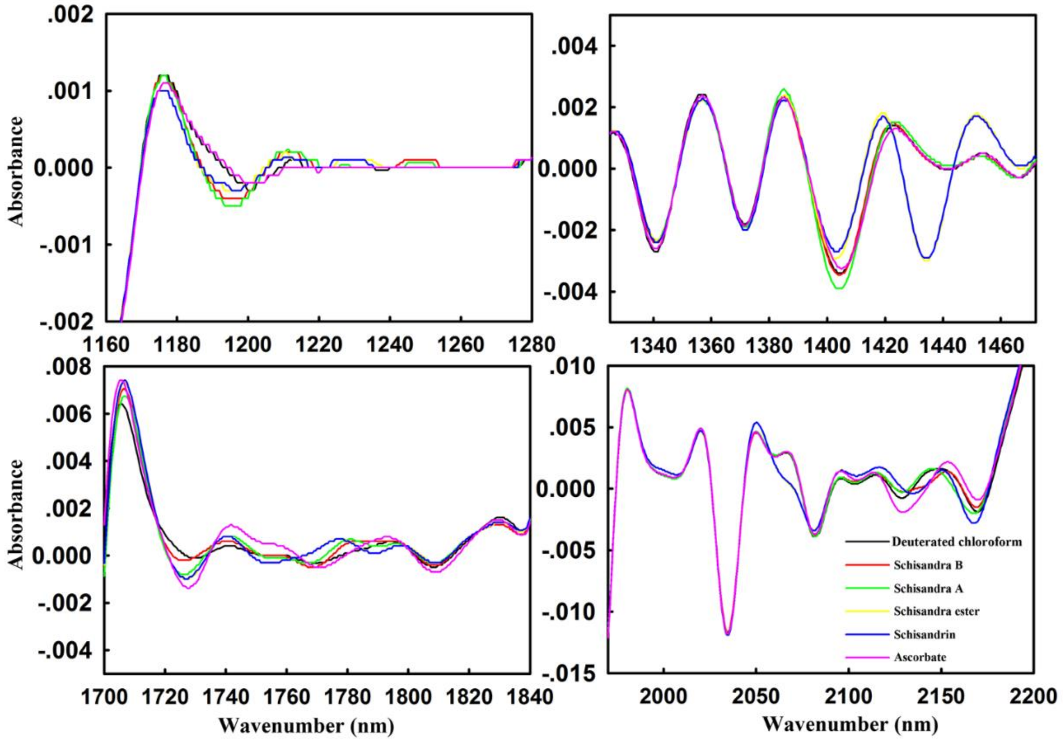

Figure S12 The partial enlargement of the 2nd spectra of lignins, including Schisandra B, Schisandra A, Schisandrin A, Schizandrol, and Ampicin.

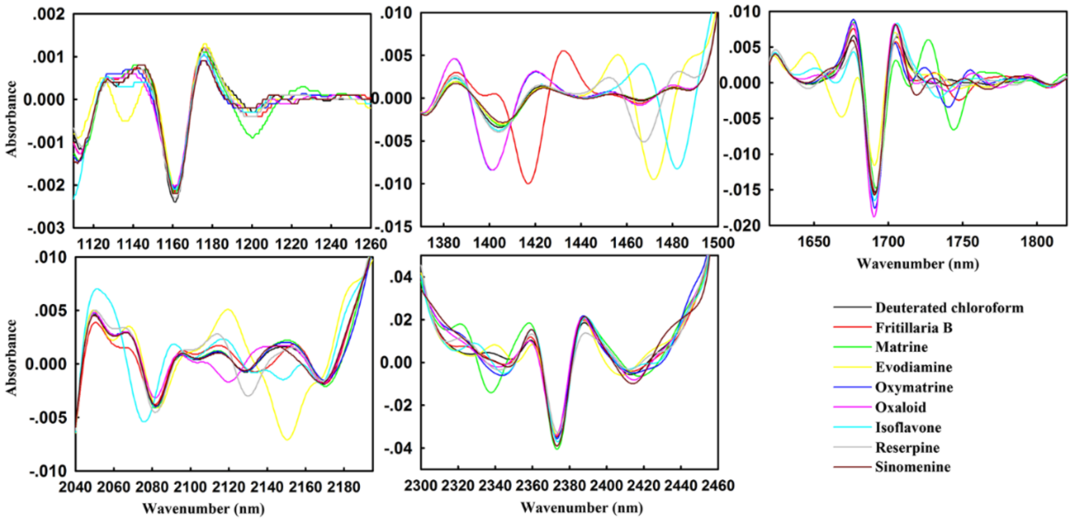

Figure S13 The partial enlargement of the 2nd spectra of alkaloids, including fritillaria B, matrine, evodiamine, oxymatrine, oxidized saponin, isorubicin, reserpine, and sinomenine.

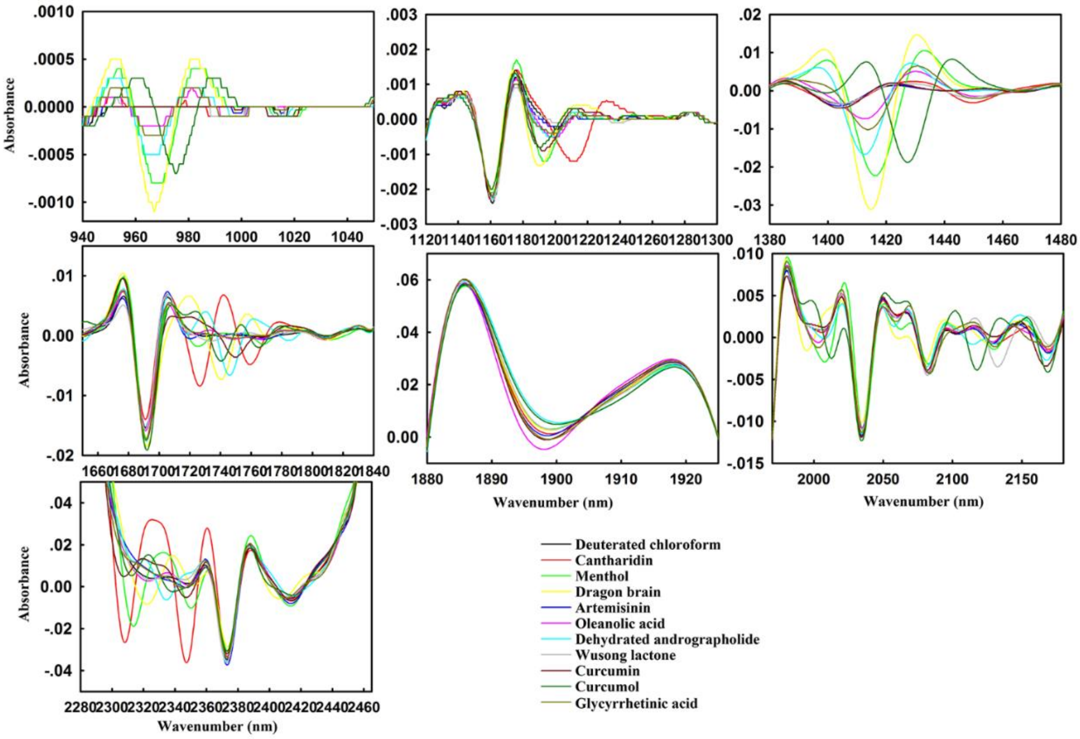

Figure S14 The partial enlargement of the 2nd spectra of terpenoids, including cantharidin, menthol, borneol, artemisinin, oleanolic acid, dehydroandrographolide, eucalyptus lactone, zedoary diketone, curcumol, and glycyrrhetinic acid.

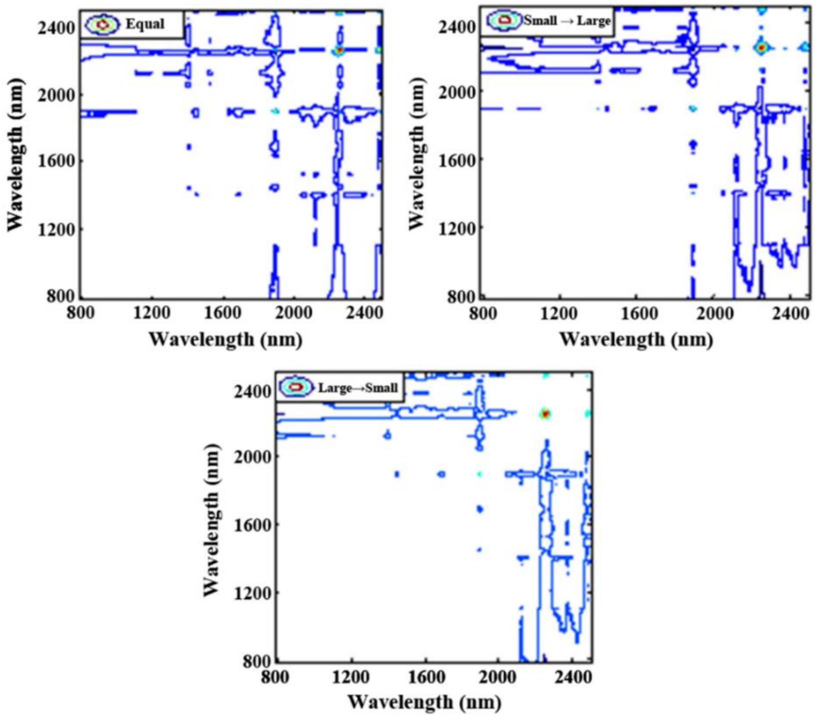

Figure S15 The synchronous 2D-COS of magnolols with different sample concentration intervals. There are 12 same autocorrelation peaks caused by this 3 kinds of sample concentration intervals at 1339 nm, 1444 nm, 1631 nm, 1690 nm, 1946 nm, 2049 nm, 2120 nm, 2286 nm, 2307 nm, 2343 nm, 2372 nm, and 2349 nm.

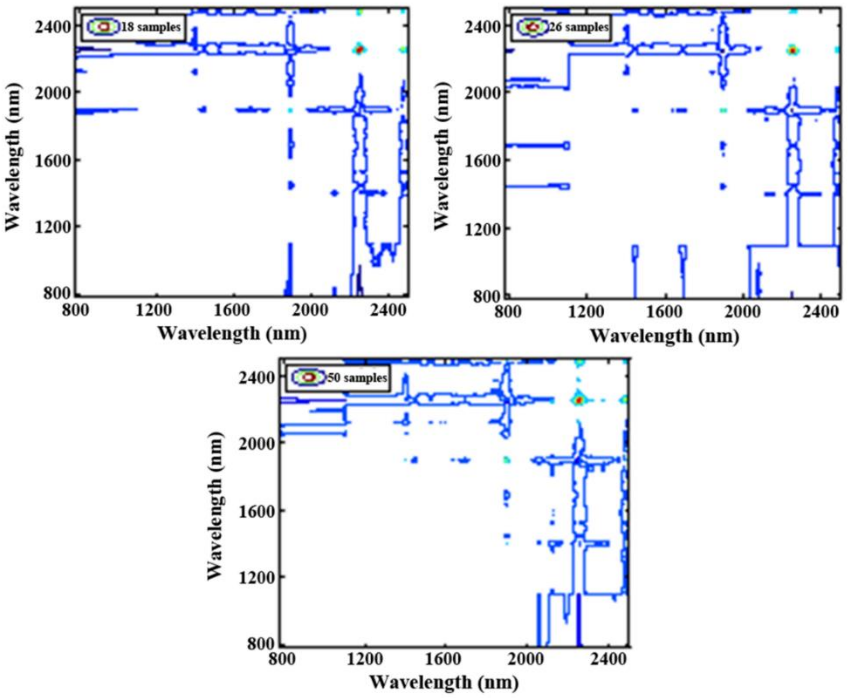

Figure S16 The synchronous 2D-COS of magnolols with different sample sizes. There are 12 same autocorrelation peaks caused by these 3 kinds of sample sizes at 1339 nm, 1444 nm, 1631 nm, 1690 nm, 1946 nm, 2049 nm, 2120 nm, 2286 nm, 2307 nm, 2343 nm, 2372 nm, and 2349 nm.

Table S1 The sample preparation for 2D-COS.

Sample name	Sample size	Specification of volumetric flask (mL)	Solvent	Sample concentration (mg*mL^{-1})
Magnolol reference substance	49.98mg	10	Deuterated chloroform solvent	1.00, 1.50, 2.00, 2.50, 3.00, 4.00, 4.50, and 5.00
Psoralen reference substance	50.04mg			1.00, 1.40, 1.80, 2.00, 2.20, 2.40, 3.50, 4.00, and 5.00
Isopsoralen reference substance	50.08mg			1.00, 1.13, 1.40, 2.00, 2.50, 3.00, 3.50, 4.00, and 5.00
Imperatorin reference substance	50.25mg			1.00, 1.13, 1.50, 2.00, 2.50, 3.00, 3.50, 4.00, and 5.03
Isoimperatorin reference substance	38.51mg			0.96, 1.12, 1.35, 1.73, 2.70, 3.08, 3.47, and 3.85
Osthole reference substance	46.85mg			0.94, 1.31, 1.62, 1.87, 3.28, 3.75, 4.22, and 4.69
Cinnamic acid reference substance	5.00mg			0.65, 1.00, 2.00, 2.50, 3.00, 3.50, 4.00, 4.50, and 5.00
Cinnamaldehyde reference substance	0.30mL			0.60, 0.81, 0.90, 1.50, 1.80, 2.10, 2.40, 2.70, and 3.00%
Eugenol reference substance	0.50mL			1.00, 2.00, 3.00, 3.50, 4.00, 4.50, 4.70, and 5.00%
Schisantherin A reference substance	51.17 mg			0.83, 1.00, 2.50, 3.00, 3.50, 4.00, 4.50, and 5.12
Schizandrin A reference substance	49.84 mg			1.00, 1.18, 2.00, 2.50, 3.00, 3.50, 4.00, 4.50, and 5.00
Schizandrin B reference substance	49.51 mg			0.63, 1.00, 2.00, 2.50, 3.00, 3.50, 4.00, 4.50, and 4.95
Sophordine reference substance	50.49 mg			1.00, 1.15, 2.00, 2.50, 3.00, 3.50, 4.00, 4.50, and 5 .00
Matrine reference substance	49.14 mg			0.68, 1.00, 2.00, 2.50, 3.00, 3.50, 4.00, 4.50, and 5 .00

Colchicine reference substance	50.77 mg	0.55, 1.00, 2.00, 2.50, 3.00, 3.50, 4.00, 4.50, and 5.01
Oxysophocaprine reference substance	52.67 mg	0.84, 1.05, 2.18, 2.64, 3.16, 3.90, 4.22, 4.73, and 5 .27
Oxymatrin reference substance	50.77 mg	0.50, 1.00, 2.00, 2.50, 3.00, 3.50, 4.00, 4.50, and 5 .01
Sinomenine reference substance	59.20 mg	0.65, 1.18, 2.37, 2.96, 3.55, 4.14, 4.74, 5.33, and 5 .92
Reserpine reference substance	49.88 mg	0.50, 1.00, 2.00, 2.50, 3.00, 3.50, 4.00, 4.50, and 5 .00
Borneol reference substance	48.36 mg	0.83, 0.97, 1.94, 2.42, 2.90, 3.39, 3.87, 4.37, and 4.84
Menthol reference substance	49.68 mg	1.00, 1.15, 2.00, 2.50, 3.00, 3.50, 4.00, 4.50, 5.00
Curcumenol reference substance	50.47 mg	0.90, 1.00, 2.00, 2.50, 3.00, 3.50, 4.00, 4.50, and 5.00
Oleanolic acid reference substance	45.26 mg	0.72, 0.91, 1.82, 2.27, 2.72, 3.17, 3.62, 4.08, and 4.53
Nor cantharidin reference substance	51.02 mg	1.00, 1.35, 2.00, 2.50, 3.00, 3.50, 4.00, 4.50, and 5.00
Artemisinin reference substance	50.47 mg	0.90, 1.00, 2.00, 2.50, 3.00, 3.50, 4.00, 4.50, and 5.00
Dehydroandrographolide reference substance	50.06 mg	0.75, 1.00, 2.00, 2.50, 3.00, 3.50, 4.00, 4.50, and 5.00
Gossypol reference substance	50.06 mg	0.2 %, 0.4 %, 0.6 %, 0.7 %, 0.8 %, 0.9 %, and 2 %
Limonin reference substance	46.04 mg	0.92, 1.84, 2.30, 2.76, 3.22, 3.68, 4.14, and 4.60

Table S2 The characteristic bands of several kinds of compounds assigned by the second derivative and 2D-COS, respectively.

Compounds	Characteristic bands (nm)	
	second derivative	2D-COS
Simple Phenylalanines	1370-1490, 1117-1285, 1560-1830, 1975-220, 2314-2470	1620-1740, 1863-1963, 2000-2500
Lignins	1160-1280, 1325-1472, 1700-1840, 1970-2200	1387-1468, 1619-1714, 1873-1912, 2018-2180, 2215-2500
Coumarins	1370-1480, 1550-1840, 2040-2200, 2301-470	1379-1415, 1595-1785, 1862-1920, 2046-2500
Terpenoids	940-1000, 1120-1300, 1380-1480, 1650-1840, 1880-1925, 1970-2180, 2280-2460	1388-1442, 1676-1761, 1864-1920, 1990-2145, 2320-2500
Alkaloids	1110-1260, 1370-1495, 1620-1820, 2040-2195, 2300-2460	1380-1428, 1656-1803, 1860-1935, 2090-2500
